# Burden of unintentional drowning in Indonesia: insights from the Global Burden of Disease Study 2019

**DOI:** 10.1136/ip-2024-045274

**Published:** 2024-08-06

**Authors:** Muthia Cenderadewi, Susan G Devine, Amy E Peden, Richard Charles Franklin, GBD 2019 Indonesia Drowning Collaborators, Qorinah Estiningtyas Sakilah Adnani

**Affiliations:** 1 College of Public Health, Medical, and Veterinary Sciences, James Cook University, Townsville, Queensland, Australia; 2 Faculty of Medicine, University of Mataram, Mataram, West Nusa Tenggara, Indonesia; 3 School of Public Health and Community Medicine, University of New South Wales, Sydney, New South Wales, Australia; 4 Royal Life Saving Society - Australia, Broadway, New South Wales, Australia

**Keywords:** Mortality, Drowning, Public Health, Health Education, Child

## Abstract

**Introduction:**

A high burden of unintentional fatal drowning has been reported in low- and middle-income countries. However, little is known about unintentional drowning in Indonesia.

**Methods:**

This population-based retrospective cohort study analysed unintentional drowning data for Indonesia sourced from The Global Burden of Disease Study 2019. Estimates of trends, mortality rates, incidence rates, years lived with disability (YLDs) and disability adjusted life years were generated.

**Results:**

A decline in unintentional drowning mortality rates was observed, with an average annual mortality rate of 2.58/100 000. Males were 1.81 (95% CI 1.79 to 1.84) times more likely than females to unintentionally drown. Average annual mortality rates were highest among the under-5 age group (9.67/100 000) and 70 and over (5.71/100 000 for males; 5.14/100 000 for females). Distributions of drowning deaths vary depending on region, with mortality rates higher in Papua, Kalimantan, Sulawesi, Maluku, Sumatra and Nusa Tenggara regions.

**Discussion:**

While a decline in drowning mortality rates in Indonesia was identified between 2005 and 2019, mortality rates for unintentional drowning remained high among children under 5 years, the elderly population and those residing in Papua, Kalimantan, Sulawesi, Maluku, Sumatra and Nusa Tenggara, warranting further focused attention.

**Conclusion:**

A downward trend in the rate of unintentional drowning deaths in Indonesia is observed from 2005 onwards, with risk variation based on age, gender and region. The findings highlight the importance of addressing drowning as a cause of premature mortality and health system burden in Indonesia, including through enhancing drowning data collection systems and identifying drowning risk factors.

**WHAT IS ALREADY KNOWN ON THIS TOPIC**

**WHAT THIS STUDY ADDS**

- Between 2005 and 2019, a decline in unintentional drowning mortality rates was observed, with an average annual mortality rate of 2.58/100 000.
- The unintentional drowning risk varies based on age, sex and region in Indonesia. Being male, aged under 5, aged 70 years and above and residing in provinces in Papua, Kalimantan, Sulawesi, Maluku, Sumatra and Nusa Tenggara, were recognised as risk factors.

**HOW MIGHT THIS STUDY AFFECT RESEARCH, PRACTICE, OR POLICY**

- The findings highlight the importance of continuing to enhance drowning data collection systems, as well as identifying drowning risk factors and developing contextualised drowning preventive strategies in Indonesia.

## Introduction

 Drowning represents a major challenge for global public health.^1 2^ In 2017, an estimated 295 210 deaths occurred globally due to unintentional drowning, with a global mortality rate of 4/100 000.^3^ Most drowning deaths worldwide occurred in low- and middle-income countries (LMICs) (91%), particularly in Southeast Asia (35%).^1^ However, less is known about unintentional drowning deaths in Indonesia, the world’s largest archipelagic state and the fourth most populated nation.^4^

**Figure 1:**
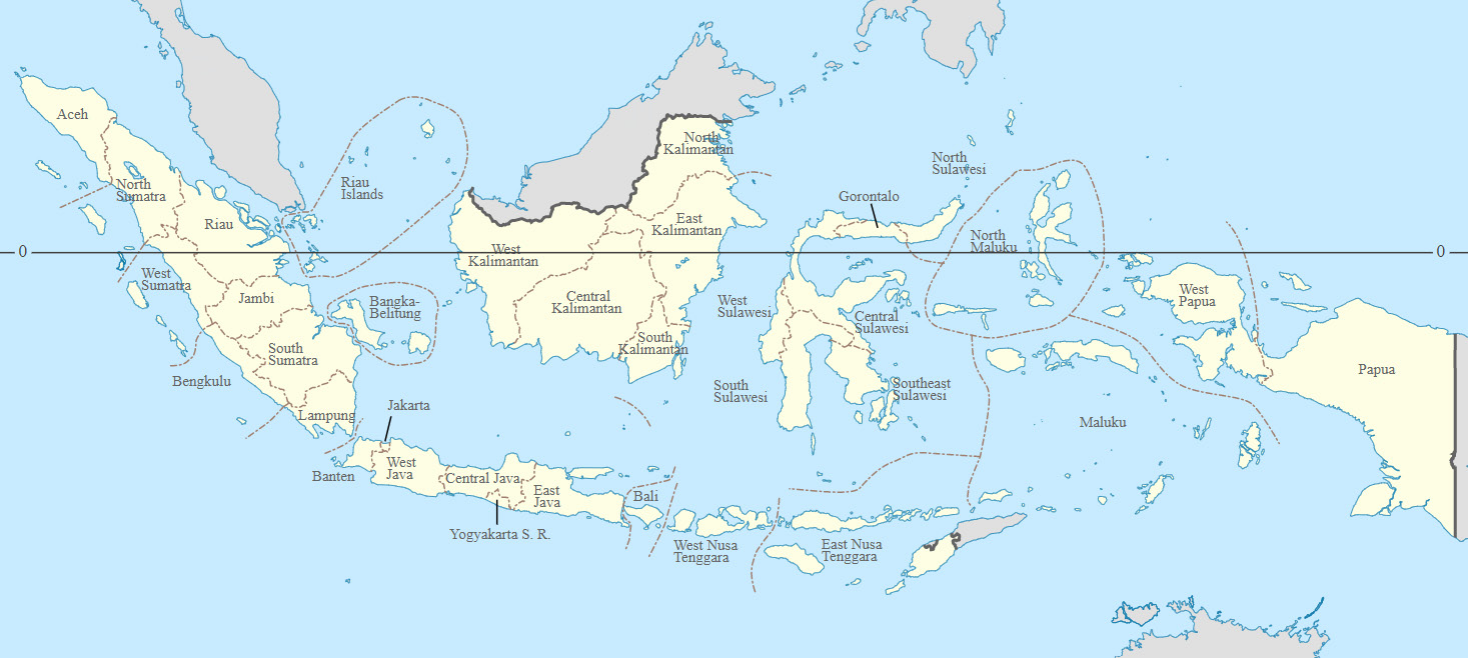
Map of Indonesia.

Located in the Southeast Asia region, Indonesia consists of 16 056 islands, with a population of over 270 200 000 and a density of 141 people/km^2^.^4^ Indonesia’s vast area comprises 1 919 440 km^2^ of land area, including 93 000 km^2^ of inland seas and 6 159 032 km^2^ of water area, exposing Indonesians to a high risk of drowning and submersion^5 6^ (figure 1). Despite this, according to the 2021 Regional Status Report on Drowning in Southeast Asia by the WHO, Indonesia does not have a national coordination mechanism for drowning prevention and water safety, and no coordinated national death registry from which national and subnational drowning data can be collected.^7^

To further understand the magnitude of drowning as a public health problem in Indonesia, this research aims to examine mortality rates, incidence rates, years of life lost (YLLs) and risk factors of fatal unintentional drowning in Indonesia and investigate the overall drowning burden via years lived with disability (YLDs) and disability adjusted life years (DALYs), between 2005 and 2019 using the 2019 Global Burden of Disease (GBD) Study estimates.

## Methods

This study was undertaken as a population-based retrospective cohort study. This study is part of a larger explanatory sequential mixed-methods study investigating unintentional drowning in Indonesia, which comprised three phases: (1) a scoping review^8^; (2) a retrospective cohort study reported here and (3) a qualitative study aimed to expand the quantitative findings. The scoping review^8^ revealed the limited availability of drowning data in Indonesia, informing our decision to use the GBD Study 2019 data as the primary source for this investigation.

An analysis of quantitative, national data sourced from the GBD Study 2019^9^ by the Institute for Health Metrics and Evaluation (IHME) database was performed to generate estimates of mortality rates, incidence rates, YLDs, YLL’s and DALYs for unintentional drowning at a national and subnational level in Indonesia, including all its 34 provinces. The data collected, spanned the period of 2005–2019 and, in coordination with the Indonesian Ministry of Health, was collected using verbal autopsy survey instruments and modelling.^9^ This current study focuses on unintentional drowning, as defined by the International Classification of Disease (ICD) ninth revision (ICD-9) and ICD-10 codes. The GBD Study 2019 used the ICD-9 code, E910 and ICD-10 codes (W65–W74) for unintentional drowning.^310^,^12^ These codes do not include unintentional drowning due to water transport and disaster, nor drowning of intentional or undetermined intent and are considered an underestimation of drowning.^13^

In this study, ‘incidence’ pertains specifically to the frequency of non-fatal drowning incidents within the Indonesian population throughout the study duration. This definition excludes drowning-related fatalities, which were treated distinctly as mortality events. Information on DALYs, YLDs and YLLs due to drowning were also inferred to assess the overall burden of drowning in Indonesia.^14^ One DALY represents the loss of an equivalence of one year of life with full health. DALYs for drowning are the sum of YLLs due to premature mortality caused by drowning and YLDs due to drowning and/or submersion.^14^

Data were downloaded using the IHME GBD results tool between March 2021 until March 2022 for collecting drowning data for the period of 2005–2019 for Indonesia and the subnational provinces.^9^ The year 2005 is chosen as the starting year of investigation, as a consensus on the establishment for a definition of drowning was issued by the WHO in 2005, which included both fatal and non-fatal drowning cases.^15^

This study complies with the Guidelines for Accurate and Transparent Health Estimates Reporting recommendations.^16^

### Data abstraction

The following data were extracted on unintentional drowning deaths and non-fatal submersion in Indonesia: mortality rates, incidence rates, YLDs, YLLs and DALYs,^14^ based on year, gender, age group (under 5, 5–14 years, 15–49 years, 50–69 years and 70+ years) and province of Indonesia.

### Analysis

Data were extracted from the GBD Study 2019 and entered into Microsoft Excel and analysed using IBM SPSS Statistics V.27. Trend analysis between the period of 2005 and 2019 was inferred with linear regression. Relative risk (RR), with a 95% CI (Confidence Interval), was calculated to measure the association between exposures of interest (sex, age group, jurisdiction or province) and unintentional drowning deaths. Where RR was calculated, the predictor group with the lowest annual mortality rate was used as the reference point (except for provinces, where the rate for Jakarta as the capital province of Indonesia was used as the reference point).

### Ethics approval

Ethics approval was granted by the University of Mataram of Indonesia (Ethics Approval number 128/UN18.F8/ETIK/2023).

### Funding

GBD is supported by the Bill and Melinda Gates Foundation. The funders of the study had no role in the study design, data collection, data analysis, data interpretation, writing of the report or the decision to submit the article for publication. All authors had full access to the data in the study and had final responsibility for the decision to submit for publication.

## Results

In total, there were 94 035 (95% UI (Uncertainty Interval): 77 135.3 to 108 737.1) unintentional drowning deaths in Indonesia between 2005 and 2019, of which 69% were males.

### Incidence and mortality rates

The average annual mortality rate in Indonesia between 2005 and 2019 was 2.58/100 000. Notably, there was a consistent decrease observed in the drowning mortality rate over this period, from 3.35/100 000 in 2005 to 1.93/100 000 in 2019 (table 1). This trend is supported by a high R^2^ of 0.99 obtained from the regression model. The regression equation (y=−0.10x+209.53) reinforces this pattern, with a negative coefficient indicating a declining trend. These results collectively suggest a significant decrease in drowning mortality rates throughout the years under analysis. While there is evidence suggesting a negative trend in non-fatal drowning incidence rates over the study period, the linear regression model fails to sufficiently explain the overall relationship between the variables (y=−0.11x+243.86, R^2^=0.06).

**Table 1:** Drowning incidence, mortality, YLLs, YLDs and DALYs in Indonesia 2005–2019

Year	Number of non-fatal drowning cases	95% UI (Uncertainty Interval)	Non-fatal drowning incidence rates (per 100 000)	95% UI (Uncertainty Interval)	Number of drowning deaths	95% UI (Uncertainty Interval)	Mortality rates (per 100 000)	95% UI (Uncertainty Interval)	YLDs	95% UI (Uncertainty Interval)	Rates of YLDs (per 100 000)	95% UI (Uncertainty Interval)	YLLs	95% UI (Uncertainty Interval)	Rates of YLLs (per 100 000)	95% UI (Uncertainty Interval)	DALYs	95% UI (Uncertainty Interval)	Rates of DALYs (per 100 000)	95% UI (Uncertainty Interval)
2005	61 352.42	45 075.22 to 84 026.33	26.98	19.82 to 36.95	7616	6202.70 to 8749.59	3.35	2.73 to 3.85	2513.69	1738.77 to 3462.23	1.11	0.76 to 1.52	542 077.91	412 084.15 to 637 426.50	238.35	181.20 to 280.28	544 591.60	414 998.38 to 639 848.26	239.46	182.48 to 281.34
2006	72 759.46	52 774.19 to 101,862.47	31.61	22.93 to 44.26	7379	5965.72 to 8428.78	3.21	2.59 to 3.66	2530.15	1733.73 to 3489.02	1.10	0.75 to 1.52	521 409.01	393 000.54 to 610 907.60	226.53	170.74 to 265.42	523 939.16	395 322.40 to 613 833.36	227.63	171.75 to 266.69
2007	60 359.17	43 904.17 to 83 543.14	25.92	18.85 to 35.87	7087	5857.66 to 8333.34	3.04	2.52 to 3.53	2536.56	1749.95 to 3504.65	1.09	0.75 to 1.50	498 411.02	385 079.04 to 592 487.36	214.02	165.36 to 254.42	500 947.59	388 154.70 to 594 943.98	215.11	166.68 to 255.47
2008	60 170.82	43 364.20 to 84 069.45	25.55	18.41 to 35.69	7051	5832.69 to 8129.72	2.99	2.48 to 3.45	2537.32	1,743,10 to 3486.62	1.08	0.74 to 1.48	495 280.81	381 643.60 to 584 417.20	210.27	162.02 to 248.11	497 818.13	384 129.77 to 586 737.39	211.34	163.08 to 249.09
2009	62 136.06	45 240.77 to 86 850.32	26.09	19.00 to 36.47	6946	5579.32 to 8011.40	2.92	2.34 to 3.36	2536.73	1747.54 to 3514.79	1.07	0.73 to 1.48	486 051.92	363,375.60 to 574,788.41	204.09	152.58 to 241.34	488 588.65	365 705.73 to 577 232.66	205.15	153.55 to 242.37
2010	63 051.52	45 705.06 to 88 193.95	26.19	18.99 to 36.64	6717	5490.60 to 7778.91	2.79	2.28 to 3.23	2536.61	1754.11 to 3498.70	1.05	0.73 to 1.45	466 659.98	352 554.59 to 551 696.60	193.87	146.47 to 229.20	469 196.59	354 600.35 to 554 989.94	194.92	147.32 to 230.57
2011	61 247.35	44 072.92 to 86 333.37	25.19	18.12 to 35.50	6520	5257.99 to 7435.72	2.68	2.16 to 3.06	2525.79	1737.92 to 3467.77	1.04	0.71 to 1.43	450 282.95	330 262.55 to 525 410.36	185.16	135.81 to 216.06	452 808.74	333 408.30 to 527 808.40	186.2	137.10 to 217.04
2012	60 870.65	43 637.47 to 85 781.12	24.79	17.77 to 34.93	6301	5139.79 to 7.239.70	2.57	2.09 to 2.95	2495.40	1722.69 to 3448.42	1.02	0.70 to 1.40	432 036.69	329 442.74 to 508.156.66	175.93	134.16 to 206.93	434 532.08	331 671.73 to 510 854.33	176.95	153.06 to 208.03
2013	60 870.50	43 636.90 to 85 568.21	24.53	17.61 to 34.52	6102	4984.85 to 7046.51	2.46	2.01 to 2.84	2460.63	1692.25 to 3390.81	0.99	0.68 to 1.37	415 522.00	312 695.14 to 490 432.54	167.64	126.16 to 197.87	417 982.62	315 094.29 to 491 253.52	168.64	127.13 to 198.60
2014	60 759.66	43 583.90 to 85 404.61	24.30	17.43 to 34.16	5878	4845.57 to 6807.30	2.35	1.94 to 2.72	2437.33	1687.56 to 3363.28	0.97	0.67 to 1.35	396 972.31	302 305.79 to 471 771.23	158.76	120.90 to 188.67	399 409.64	304 534.71 to 474 405.89	159.73	121.79 to 189.73
2015	60 851.40	43 576.42 to 85 456.30	24.13	17.28 to 33.89	5630	4621.39 to 6562.34	2.23	1.83 to 2.60	2440.46	1673.68 to 3373.42	0.97	0.66 to 1.34	377 641.95	293 571.22 to 450 819.91	149.78	116.44 to 178.80	380 082.41	295 685.12 to 453 071.62	150.75	117.27 to 179.70
2016	63 769.57	45 682.88 to 89 662.94	25.10	17.98 to 35.28	5393	4492.61 to 6231.97	2.12	1.77 to 2.45	2602.20	1799.32 to 3593.91	1.02	0.72 to 1.41	356 334.33	279 104.49 to 423 869.44	140.23	109.83 to 166.80	358 936.53	281 762.31 to 426 339.69	141.25	110.88 to 167.68
2017	65 971.37	47 182.44 to 93 070.90	25.77	18.43 to 36.36	5251	4365.57 to 6.158.09	2.05	1.71 to 2.41	2795.01	1928.99 to 3847.61	1.09	0.75 to 1.50	343 360.19	263 368.63 to 410 262.08	134.13	102.88 to 160.27	346 155.20	266 125.58 to 412 660.11	135.22	103.96 to 161.20
2018	76 593.68	55 309.66 to 107 131.58	29.72	21.46 to41.56	5153	4396.00 to 6089.18	2	1.67 to 2.36	2866.57	1975.54 to 3940.32	1.1	0.77 to 1.53	334 335.24	264 447.55 to 401 656.57	129.71	102.59 to 155.83	337 201.81	266 977.83 to 404 131.29	130.82	103.58 to 156.79
2019	67 737.79	48 714.70 to 95 161.42	26.11	18.77 to 36.68	5011	4192.79 to 5845.54	1.93	1.62 to 2.25	2925.14	2026.13 to 4049.23	1.13	1.44 to 2.74	321 737.24	256 585.39 to 381 494.75	124	98.8 to 147.03	324 662.37	258 835.12 to 384 151.12	125.13	99.76 to 148.05

DALYs, disability-adjusted life years; YLDs, years of living with disability; YLLs, years of life lost.

### Mortality rates by age group and gender

**Figure 2:**
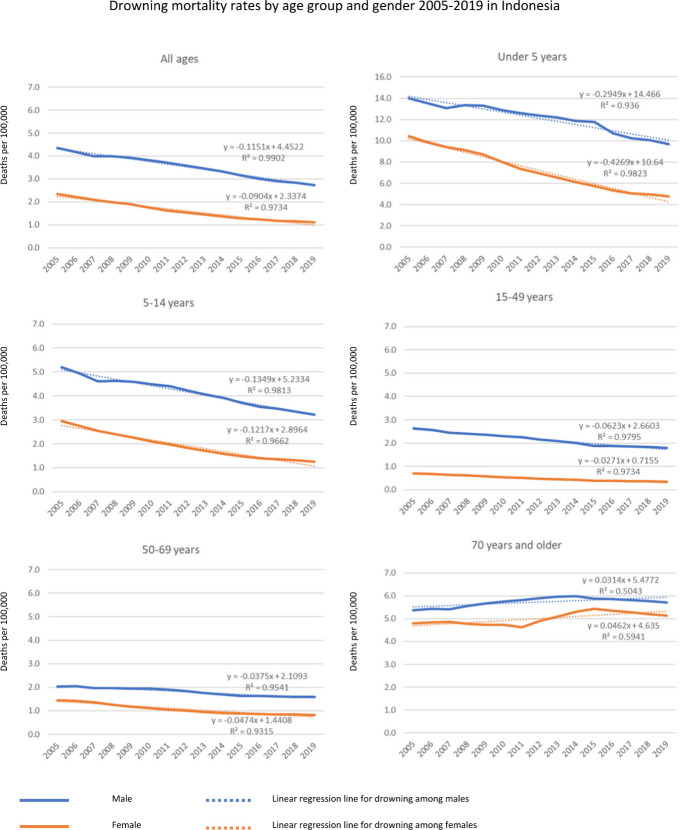
Unintentional drowning mortality rates by age group and gender in Indonesia 2005–2019

Between 2005 and 2019, drowning mortality rates for both males and females of all ages decreased in Indonesia (figure 2 and online supplemental table S2). The highest drowning mortality rate across the 15-year period was identified among under-5 males, with an average annual mortality rate of 9.67/100 000 between 2005 and 2019, contributing the largest proportion of deaths by unintentional drowning in Indonesia (34.74%) (figure 2, online supplemental table S1). Between 2005 and 2019, unintentional drowning mortality rates were higher for males than females across all age groups (figure 2, table 2, online supplemental table S2).

**Table 2:** Risk factors of fatal unintentional drowning in Indonesia

	Total deaths by unintentional drowning (n=94 035)	Average annual mortality rate per 100 000 (between 2005 and 2019)	Relative risk (RR)	95% CI
Sex				
Male	64 898	5.227	1.81	1.79 to 1.84
Female*	29 137	3.142	1*	Ref
Age group				
Under 5	32 663	9.665	3.67	3.63 to 3.72
5–14 years	21 222	3.038	1.97	1.94 to 2.00
15–49 years*	26 892	1.331	1*	Ref
50–69 years	7149	1.436	0.87	0.85 to 0.89
70+ years	6109	5.367	2.50	2.45 to 2.56
Jurisdictions/provinces				
Aceh	2082	2.998	1.63	1.54 to 1.73
Bali	868	1.464	0.84	0.78 to 0.91
Bangka-Belitung Islands	568	2.972	1.60	1.47 to 1.76
Banten	4576	2.790	1.49	1.42 to 1.56
Bengkulu	668	2.490	1.41	1.30 to 1.54
Central Java	10 141	2.051	1.23	1.18 to 1.28
Central Kalimantan	2382	6.783	3.69	3.49 to 3.90
Central Sulawesi	874	2.133	1.20	1.12 to 1.30
East Java	11 619	2.062	1.23	1.18 to 1.29
East Kalimantan	993	2.076	1.12	1.04 to 1.21
East Nusa Tenggara	3115	4.286	2.40	2.28 to 2.53
Gorontalo	804	4.899	2.81	2.60 to 3.04
Jakarta*	2510	1.755	1*	Ref
Jambi	1468	3.066	1.70	1.60 to 1.82
Lampung	2778	2.365	0.73	0.69 to 0.78
Maluku	1198	5.004	2.79	2.61 to 3.00
North Kalimantan	623	7.226	3.53	3.23 to 3.85
North Maluku	769	4.808	2.57	2.38 to 2.79
North Sulawesi	470	1.393	0.79	0.71 to 0.87
North Sumatra	8137	4.062	2.35	2.25 to 2.46
Papua	3203	6.918	3.98	3.781 to 4.20
Riau	2085	2.370	1.26	1.19 to 1.33
Riau Islands	529	2.050	1.02	0.93 to 1.12
South Kalimantan	1893	3.286	1.88	1.77 to 1.99
South Sulawesi	4511	3.666	2.14	2.04 to 2.25
South Sumatra	4249	3.701	2.11	2.01 to 2.22
Southeast Sulawesi	988	2.835	1.54	1.43 to 1.65
West Java	12 442	1.857	1.06	1.02 to 1.11
West Kalimantan	1557	2.271	1.29	1.21 to 1.38
West Nusa Tenggara	2443	3.520	2.03	1.92 to 2.14
West Papua	397	3.350	1.74	1.57 to 1.93
West Sulawesi	837	4.605	2.55	2.36 to 2.76
West Sumatra	1435	1.940	1.11	1.04 to 1.18
Yogyakarta	824	1.573	0.33	0.30 to 0.36

* Where RR was calculated, the group with the lowest annual mortality rate (except for jurisdictions, where the rate for Jakarta was used) for unintentional drowning deaths was used as the reference point.

### Mortality rates by province

**Figure 3:**
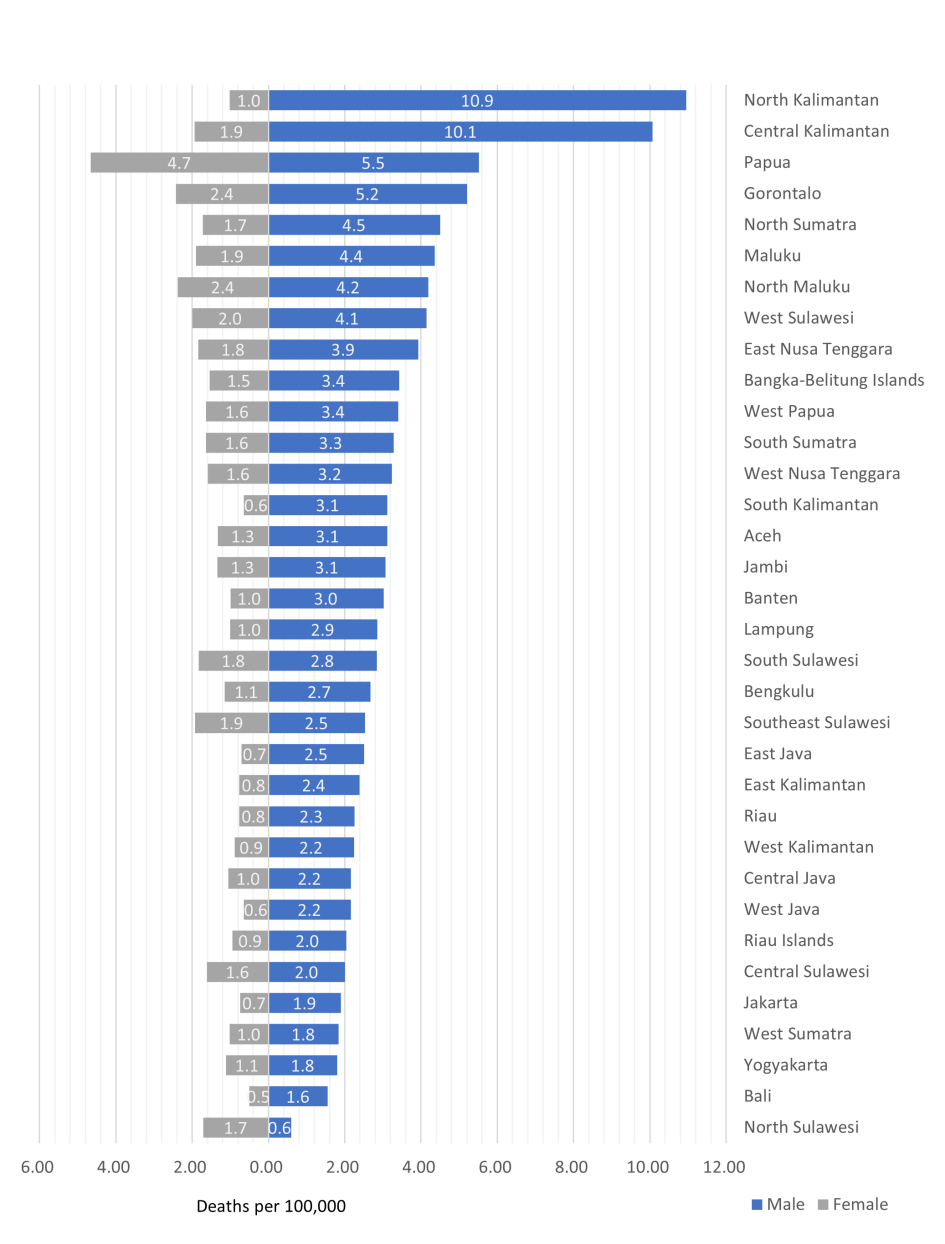
The 2019 mortality rates of unintentional drowning of all ages by sex and province in Indonesia.

Distributions of drowning deaths by sex vary depending on region. Of 34 provinces in Indonesia, the highest drowning death rates for all age groups in the year 2019 were observed in male populations in the provinces of North Kalimantan (10.95/100 000), Central Kalimantan (10.06/100 000), Papua (5.51/100 000 populations) and Gorontalo (5.21/100 000), which are located in the central and eastern part of Indonesia, in comparison with mortality rates from unintentional drowning in other provinces in the western part of Indonesia (figure 3, online supplemental table S1).

**Figure 4:**
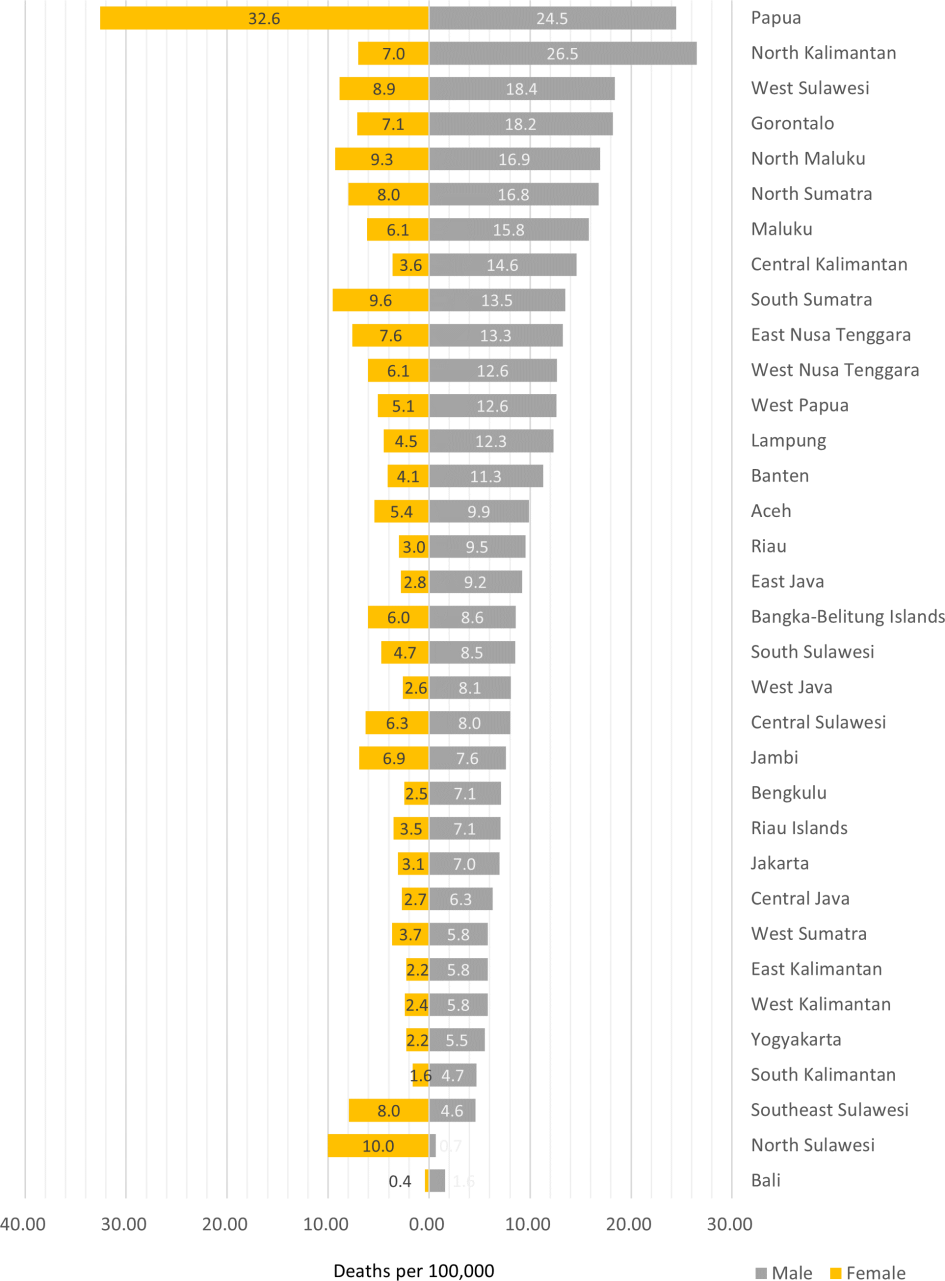
The 2019 under-5 unintentional drowning mortality rates by sex and province in Indonesia.

In 2019, the highest male under-5 drowning mortality rates were observed in North Kalimantan (26.50/100 000), Papua (24.46/100 000) and West Sulawesi (18.38/100 000) (figure 4 and online supplemental table S1). For female populations, the highest drowning death rates for the under-5 age group in the year 2019 were observed in the province of Papua (32.58/100 000), which was higher than in other provinces (figure 4 and online supplemental table S1). Between 2005 and 2019, several provinces experienced the highest reductions in child drowning cases, including Maluku (y=−1.44x+30.51, R²=0.97), West Nusa Tenggara (y=−0.99x+23.35, R²=0.98), Papua (y=−0.92x+42.45, R²=0.92), West Sulawesi (y=−0.97 x+27.19, R²=0.98), North Maluku (y=−0.86x+25.07, R²=0.98), South Sulawesi (y=−0.85x+18.42, R²=0.98), East Nusa Tenggara (y=−0.83x+21.37, R²=0.96), South Sumatra (y=−0.81x+22.90, R²=0.98), North Kalimantan (y=−0.72x+27.42, R²=0.94) and Riau (y=−0.61x+15.34, R²=0.96).

### YLDs and DALYs

Unintentional drowning DALYs showed a decrease between 2005 and 2019 (y=−8.26x+243.95, R^2^=0.99), with rate of DALYs of 239.46 (95% UI: 182.48 o 281.34) in 2005 and 125.13 (95% UI: 99.76 to 148.05) in 2019 (table 1).

### Risk factors

In Indonesia, males were 1.81 times (95% CI 1.79 to 1.84) more likely than females to unintentionally drown (table 2). Indonesian children aged less than 5 years old were 3.67 times (95% CI 3.63 to 3.72) more likely to become victims of fatal drowning in comparison to individuals aged between 15 and 49 years (table 2). Elderly populations were also an important contributor, with individuals aged 70 years and above 2.5 times (95% CI 2.45 to 2.56) more likely to fatally drown in comparison to individuals aged 15–49 years (table 2).

The top three highest average annual mortality rates for unintentional drowning were registered in the provinces of North Kalimantan (7.23/100 000), Papua (6.92/100 000) and Central Kalimantan (6.78/100 000), and individuals in Papua had the highest likelihood of dying from unintentional drowning (RR=3.98), compared with the reference group of the metropolitan capital of Indonesia, Jakarta (table 2)

## Discussion

Unintentional drowning is a little studied public health issue in Indonesia. Overall, this study identifies a decline in drowning mortality rates in Indonesia between 2005 and 2019 (R^2^=0.99, y=−0.10x+209.53). During the 15-year study period, mortality rates for unintentional drowning were higher in males than females and also higher among children aged under 5 years, elderly populations aged 70 years and above, and populations residing in the Papua, Kalimantan, Sulawesi, Maluku, Sumatra and Nusa Tenggara regions. These findings underscore the need for further focused attention and interventions in these demographic groups and geographical areas.

### The rates and trends of unintentional drowning in Indonesia

Overall, there was a decrease in drowning mortality rates, with an average annual mortality rate between 2005 and 2019 of 2.58/100 000. However, it is acknowledged that the GBD Study 2019 data for Indonesia was mostly sourced from verbal autopsy data, and only reported unintentional drowning, while excluding cases caused by water-transport related and disaster-related drowning incidents, thus potentially underrepresenting the actual magnitude of drowning in Indonesia. A previous study in Australia has reported how different ICD-10 coding combinations affected the capture of drowning deaths in the national register.^13^ When specific ICD codes of W65–W74 for accidental drowning and submersion were used, as in the GBD Study 2019, only 61% of unintentional drowning deaths were captured. However, inclusion of additional drowning-related codes for accidental drowning related to watercrafts, floodings and undetermined intent increased the capture rate to 78%, and when the drowning codes used were expanded to include intentional drowning events, with multiple causes of death considered, the capture rate rose to 92%.^13^

DALYs attributed to unintentional drowning in Indonesia declined between 2005 and 2019 (table 1 and online supplemental table S1). The observed decrease in incidents of drowning among children under the age of 5 in Indonesia during the study period (y=−0.36x+740.01, R^2^=0.98) likely contributes to the overall reduction in DALYs. In this study, the low YLDs correspond with findings from a previous study, which reported lower YLDs for children aged under 5 in LMICs, compared with high-income nations. This is attributed to the higher proportion of fatal drownings in LMICs.^17^

### The risk of drowning among males in Indonesia: informing preventive measures

This study found that in Indonesia, males were 1.81 times (95% CI 1.79 to 1.84) more likely than females to unintentionally drown. Among high-income nations, a common observation is the higher likelihood of males experiencing unintentional drowning, which has been linked to risky behaviours. This includes males tending to underestimate the risk of experiencing unintentional drowning and overestimate their knowledge and skill in water-related activities.^18^ Therefore, further research on the contributing factors and protective factors related to the risk of drowning is crucial. These factors may encompass behavioural and sociocultural aspects of drowning and are important in understanding drowning prevention suitable for the Indonesian context.

### Unintentional drowning as a leading cause of injury death for Indonesian children

Indonesian children aged under 5 years were 3.67 times (95% CI 3.63 to 3.72) more likely to die from unintentional drowning compared with populations aged 15–49 years. Under-5 drowning mortality rates in Indonesia vary across regions. For instance, in Papua, the under-5 mortality rate in 2019 was 32.6/100 000 for females and 25.4/100 000 for males, surpassing those of other provinces in the country. This finding corresponds to the 2014 WHO Global Report on Drowning which showed children aged under 5 years being disproportionately at risk for drowning.^1^ This underscores the urgent need for tailoring drowning prevention strategies in Indonesia to effectively address the heightened risk of drowning among children under the age of 5, particularly across rural populations of eastern Indonesia.

There is limited understanding on contributing factors to the observed decline in child drowning rates in Indonesia.^8^ However, the advancement of socioeconomic determinants of health, particularly the rise in gross domestic product (GDP) per capita, educational attainments and healthcare expenditure, has been identified as an instrumental driver to the reduction of drowning prevalence, including in under-5 populations, worldwide.^12^ This highlights the critical need for further investigation into how socioeconomic advancements and implemented interventions can effectively mitigate the burden of child drowning fatalities across Indonesia.

### Fatal unintentional drowning among elderly Indonesians

Individuals aged 70 years and above were 2.5 times more likely to fatally drown compared with individuals aged 15–49 years (table 2). This finding corresponds to reported higher mortality rates among older populations in other countries, including Japan, China, Australia, Canada and New Zealand.^19 20^ From 1950 to 2021, the average global life expectancy at birth has risen by 22.7 years,^21^ and this prolonged lifespan may contribute to the concurrent increase in drowning-related fatalities among older age groups. However, efforts to reduce drowning among older populations have lagged behind that of young children.^20^ The findings of the current study should be a call to action to invest in drowning prevention among older people in Indonesia.

### Jurisdiction as a determinant for unintentional drowning in Indonesia

The distribution of drowning deaths across Indonesia exhibits regional disparities, with the highest mortality rates recorded in the provinces of North Kalimantan (7.23/100 000), Papua (6.92/100 000) and Central Kalimantan (6.78/100 000). This discrepancy underscores the crucial need to investigate how socioeconomic determinants, infrastructure investments and social and environmental changes influence drowning fatalities. Particularly notable are provinces in Kalimantan, Papua, Sulawesi, Maluku and Nusa Tenggara regions, which present some of the nation’s lowest GDP per capita, alongside the highest rates of drowning mortality and the highest reductions of child drowning mortalities throughout the 15-year study period.^22^ Therefore, it is imperative to evaluate the availability and effectiveness of water safety promotion strategies and drowning prevention interventions at both national and provincial levels in Indonesia and their impact on the varying mortality rates across provinces, particularly in provinces that have experienced the highest reductions in Maluku, Nusa Tenggara, Papua, Sulawesi and Kalimantan.

### Recommendations

#### Future research

While this study has provided insight into the issue of unintentional drowning in Indonesia, several key areas for future research are noted: (1) comprehensive examination of mortality and burden associated with all ICD codes for drowning, encompassing unintentional drowning, water transport-related drowning, disaster-related drowning, drowning of undetermined intent and intentional drowning; (2) investigation of drowning risk factors specific to Indonesia and its individual provinces; (3) exploration of the interconnectedness between drowning prevention efforts and initiatives aimed at improving social determinants of health and (4) evaluation of the availability and effectiveness of water safety promotion and drowning prevention interventions.

#### Policy development

The study underscores the urgent need to advance drowning prevention efforts through robust data collection to inform burden and risk factor identification, as well as agenda setting.^23^ Immediate measures are required to strengthen the capabilities of the Indonesian public health system, establish standardised national reporting structures for health and mortality data, foster collaboration across multiple sectors and secure political and financial investment to construct an integrated drowning data collection system in Indonesia. In addition, the study emphasises the urgent need to tailor drowning prevention strategies in Indonesia to effectively address the heightened risk of drowning among children under 5 years of age, particularly in rural populations across eastern Indonesia.

#### Practice

The increased risk of drowning among children under 5 years of age emphasises the importance of adopting WHO-recommended prevention strategies aimed at reducing drowning fatalities in younger children, including enhancing supervision, establishing community-based childcare centres and installing barriers to limit children’s access to water bodies.^23 24^ However, effective implementation of these interventions requires tailoring to local contexts to ensure the effectiveness and sustainability of drowning prevention efforts in reducing child drowning fatalities in Indonesia.^25^,^27^

### Strengths and limitations

This is the first study to explore the epidemiology of drowning in Indonesia. A key strength of this study is the mutually exclusive and exhaustively collected data available via the GBD Study, for both ICD-9 and ICD-10 coded cases for different time periods.^3 11 12^

However, as in many cases of less optimal injury surveillance systems in developing nations, including in Indonesia, the data on drowning as a cause of death has been collected from verbal autopsy survey instruments, which may result in the underestimation of the actual number of unintentional drowning cases in Indonesia.^10 11^ Moreover, the GBD Study 2019 only reported accidental drowning and submersion events (coded by ICD-10 as W65–W74), excluding disaster-related and water transport-related incidents, which may further limit understanding of the magnitude of drowning in Indonesia, where hydrometeorological disasters and water transport-related injuries frequently take place.^13^

## Conclusions

Between 2005 and 2019, there was a downward trend in the rate of drowning deaths in Indonesia. Being male, aged under 5 years, aged 70 years and above and residing in provinces of Kalimantan, Papua, Sulawesi, Maluku, Sumatra and Nusa Tenggara regions, were recognised as risk factors. The findings highlight the importance of continuing to enhance data collection systems, identifying risk factors and developing contextualised preventive strategies for drowning in Indonesia.

## Supplementary material

10.1136/ip-2024-045274 online supplemental file 1

## Data Availability

All data relevant to the study are included in the article or uploaded as supplementary information.
