# Supplementary material for: Burden of unintentional drowning in Indonesia: insights from the Global Burden of Disease Study 2019
Source: Inj Prev. 2024 Aug 6;31(6):e045274. doi: 10.1136/ip-2024-045274 (PMC12703239; doi:10.1136/ip-2024-045274)
Supplement: online supplemental file 1 [file ip-31-6-s001.pdf]

Supplementary Tables

Supplementary Table S1. Mortality rates of unintentional drowning by sex, age group and province in Indonesia between 2005 and 2019

Year	Jurisdictions	Mortality rates (per 100,000)											
		Under 5		5-14 years		15-49 years		50-69 years		70+ years		All Ages	
		Male	Female	Male	Female	Male	Female	Male	Female	Male	Female	Male	Female
**2005**	Aceh	16.039	13.927	6.034	3.189	2.832	0.545	1.811	1.032	6.937	6.007	5.136	2.830
	Bali	5.949	2.332	3.235	0.894	2.545	0.396	2.759	0.959	4.832	2.812	3.095	0.826
	Bangka-Belitung Islands	8.691	9.591	4.731	3.148	4.133	1.281	2.292	2.143	7.384	5.766	4.604	2.736
	Banten	18.781	9.981	7.012	2.930	2.652	0.595	1.577	1.019	6.301	6.610	5.275	2.242
	Bengkulu	12.311	6.607	5.120	2.708	2.850	1.055	1.711	1.392	5.224	6.522	4.348	2.183
	Central Java	7.055	6.787	3.043	3.211	2.168	0.797	2.387	2.142	4.214	5.516	2.896	2.158
	Central Kalimantan	19.038	8.008	14.214	3.874	11.198	1.425	12.530	3.323	23.715	14.111	12.989	3.024
	Central Sulawesi	3.103	13.571	0.778	4.707	1.092	1.328	1.109	2.157	1.873	4.833	1.282	3.660
	East Java	13.405	5.348	5.027	1.913	2.396	0.453	1.778	1.044	6.148	3.400	3.823	1.295
	East Kalimantan	10.416	5.794	4.162	1.484	2.739	0.551	1.826	1.282	5.811	6.740	3.849	1.495
	East Nusa Tenggara	23.750	19.566	10.421	6.083	3.649	0.975	2.342	1.588	5.537	4.796	8.035	4.721
	Gorontalo	21.765	13.266	12.564	7.181	3.627	2.194	2.758	3.308	4.317	6.124	7.543	4.647
	Jakarta	10.422	6.464	4.017	1.841	2.009	0.434	1.486	0.834	6.158	6.293	3.190	1.394
	Jambi	9.694	13.395	6.807	1.495	6.590	0.955	5.342	1.359	11.815	7.925	6.935	2.535
	Lampung	12.655	7.123	5.288	2.024	2.292	0.311	1.582	0.878	6.260	6.123	4.039	1.584
	Maluku	36.404	25.145	9.915	6.506	3.238	1.914	2.415	2.316	4.861	5.515	9.326	6.176
	North Kalimantan	40.665	15.850	10.632	2.042	10.568	0.314	17.242	0.513	35.387	2.547	15.150	2.757
	North Maluku	25.003	25.703	9.514	7.056	3.203	2.289	2.088	3.148	4.835	6.709	7.440	6.509
	North Sulawesi	0.722	14.386	0.673	2.528	0.737	0.664	1.099	1.346	1.674	7.081	0.794	2.642

	North Sumatra	20.465	14.110	8.118	3.803	3.829	0.865	2.659	1.697	7.264	7.111	6.912	3.364
	Papua	30.656	55.952	8.876	4.900	3.322	0.976	2.157	1.583	6.593	7.550	8.336	9.628
	Riau	19.300	9.119	5.219	2.444	1.540	0.402	1.281	0.904	5.357	6.183	4.587	2.040
	Riau Islands	12.673	8.384	5.298	3.115	1.767	0.501	1.051	1.018	5.117	6.778	3.647	2.020
	South Kalimantan	21.368	2.888	12.110	1.043	9.807	0.352	3.514	0.252	15.282	0.957	10.959	0.749
	South Sulawesi	17.910	16.729	7.852	6.726	1.911	2.885	1.568	5.928	2.652	14.485	5.111	5.886
	South Sumatra	19.781	24.438	6.945	4.546	3.664	0.922	2.884	0.771	8.147	2.618	6.149	4.231
	Southeast Sulawesi	5.071	14.136	3.795	4.670	3.091	1.473	1.852	2.214	3.749	5.448	3.421	3.941
	West Java	11.360	5.926	3.247	1.833	1.877	0.387	1.492	0.581	4.361	2.224	3.176	1.319
	West Kalimantan	14.813	10.109	4.945	2.676	2.613	0.626	1.899	1.170	6.347	6.344	4.519	2.319
	West Nusa Tenggara	19.153	26.834	4.570	6.827	1.798	1.016	1.639	1.711	2.830	3.568	4.465	5.070
	West Papua	19.333	13.785	5.486	3.037	2.100	1.615	1.917	2.697	4.527	6.181	5.109	3.685
	West Sulawesi	31.432	22.496	9.799	5.060	2.895	0.904	1.988	1.674	6.056	7.770	8.466	4.872
	West Sumatra	9.074	11.454	2.697	1.707	1.353	0.610	1.244	0.832	2.171	1.795	2.584	2.106
	Yogyakarta	5.153	2.632	1.969	0.913	1.479	0.465	1.387	1.309	4.929	7.094	2.015	1.255
	**Indonesia**	**13.985**	**10.451**	**5.213**	**2.959**	**2.633**	**0.715**	**2.035**	**1.441**	**5.383**	**4.794**	**4.349**	**2.340**
**2006**	Aceh	15.324	12.875	5.717	2.914	2.763	0.532	1.844	1.065	7.028	6.181	4.928	2.639
	Bali	5.624	2.122	2.955	0.801	2.392	0.369	2.681	0.940	4.784	2.785	2.914	0.774
	Bangka-Belitung Islands	8.550	9.083	4.565	2.858	3.967	1.207	2.250	2.114	7.320	5.804	4.447	2.573
	Banten	17.909	9.302	6.547	2.667	2.585	0.564	1.611	1.011	6.441	6.778	5.026	2.089
	Bengkulu	11.855	6.128	4.817	2.480	2.773	1.020	1.722	1.384	5.296	6.615	4.176	2.055
	Central Java	6.945	6.495	2.922	3.028	2.141	0.774	2.425	2.143	4.329	5.618	2.853	2.091
	Central Kalimantan	18.330	7.487	13.312	3.516	10.841	1.357	12.735	3.236	24.121	14.153	12.533	2.841
	Central Sulawesi	3.030	12.537	0.733	4.314	1.054	1.261	1.096	2.123	1.893	4.851	1.240	3.401
	East Java	13.054	5.104	4.781	1.762	2.319	0.426	1.775	1.009	6.198	3.410	3.698	1.231
	East Kalimantan	9.770	5.274	3.972	1.381	2.761	0.538	1.815	1.258	5.722	6.705	3.747	1.403
	East Nusa Tenggara	22.299	17.973	9.743	5.608	3.465	0.929	2.319	1.587	5.593	4.925	7.557	4.378
	Gorontalo	21.121	12.518	11.914	6.795	3.542	2.145	2.775	3.310	4.384	6.200	7.248	4.449

	Jakarta	9.974	6.035	3.824	1.708	1.932	0.410	1.463	0.807	6.101	6.233	3.056	1.308
	Jambi	9.419	13.288	6.509	1.384	6.621	0.940	5.477	1.349	11.914	7.963	6.874	2.487
	Lampung	12.591	6.905	5.069	1.965	2.251	0.311	1.572	0.874	6.285	6.235	3.943	1.546
	Maluku	33.889	22.819	9.350	5.905	3.201	1.828	2.473	2.303	5.030	5.565	8.810	5.672
	North Kalimantan	37.673	14.293	10.013	1.888	10.295	0.297	17.387	0.504	35.671	2.556	14.489	2.485
	North Maluku	23.769	23.808	8.916	6.410	3.030	2.190	2.064	3.138	4.872	6.781	7.009	6.041
	North Sulawesi	0.704	13.519	0.647	2.432	0.706	0.643	1.078	1.339	1.663	7.111	0.770	2.518
	North Sumatra	19.820	13.308	7.685	3.458	3.718	0.807	2.647	1.645	7.305	7.107	6.640	3.139
	Papua	29.928	53.364	8.457	4.571	3.168	0.914	2.150	1.549	6.582	7.563	8.024	9.107
	Riau	18.638	8.583	4.918	2.226	1.497	0.380	1.283	0.882	5.395	6.180	4.398	1.909
	Riau Islands	11.969	7.796	5.015	2.880	1.694	0.494	1.060	1.051	5.192	6.981	3.488	1.915
	South Kalimantan	20.017	2.716	11.215	0.975	9.396	0.334	3.474	0.254	15.197	0.982	10.363	0.705
	South Sulawesi	17.486	15.629	7.345	6.093	1.838	2.715	1.577	5.767	2.707	14.228	4.876	5.510
	South Sumatra	19.165	22.998	6.657	4.388	3.558	0.892	2.849	0.776	8.067	2.677	5.932	4.002
	Southeast Sulawesi	4.984	13.442	3.618	4.332	2.978	1.397	1.843	2.182	3.725	5.484	3.303	3.723
	West Java	11.013	5.618	3.090	1.700	1.810	0.369	1.493	0.578	4.434	2.292	3.057	1.245
	West Kalimantan	13.776	9.204	4.568	2.419	2.499	0.594	1.891	1.164	6.357	6.395	4.241	2.134
	West Nusa Tenggara	18.206	24.340	4.276	6.244	1.738	0.960	1.627	1.669	2.850	3.590	4.234	4.620
	West Papua	19.686	13.677	5.996	3.540	2.603	1.817	1.952	2.777	4.490	6.236	5.523	3.897
	West Sulawesi	30.291	21.200	9.224	4.762	2.779	0.894	1.957	1.694	5.995	7.848	8.055	4.606
	West Sumatra	8.672	10.612	2.496	1.537	1.315	0.578	1.249	0.826	2.199	1.809	2.460	1.949
	Yogyakarta	5.065	2.542	1.908	0.861	1.447	0.438	1.388	1.275	4.964	7.057	1.982	1.225
	**Indonesia**	**13.518**	**9.855**	**4.937**	**2.741**	**2.559**	**0.682**	**2.042**	**1.418**	**5.443**	**4.834**	**4.191**	**2.212**
**2007**	Aceh	14.755	11.927	5.406	2.665	2.727	0.520	1.888	1.094	7.162	6.375	4.760	2.469
	Bali	5.542	2.030	2.787	0.749	2.297	0.355	2.621	0.921	4.773	2.773	2.812	0.747
	Bangka-Belitung Islands	8.486	8.793	4.377	2.744	3.894	1.123	2.219	1.978	7.291	5.634	4.347	2.448
	Banten	17.302	8.806	6.093	2.411	2.507	0.531	1.636	0.998	6.607	6.968	4.806	1.958
	Bengkulu	11.469	5.751	4.545	2.287	2.707	0.981	1.727	1.371	5.393	6.744	4.026	1.945
	Central Java	6.844	6.426	2.682	2.783	1.796	0.628	2.061	1.782	3.852	5.000	2.535	1.877

	Central Kalimantan	17.724	6.741	12.495	3.132	10.575	1.537	12.892	4.091	24.346	15.148	12.154	2.868
	Central Sulawesi	2.779	11.707	0.688	3.974	0.992	1.145	1.060	1.955	1.848	4.698	1.158	3.133
	East Java	12.815	4.949	4.562	1.641	2.260	0.412	1.773	1.010	6.278	3.527	3.594	1.197
	East Kalimantan	9.227	4.855	3.839	1.307	2.811	0.530	1.808	1.236	5.642	6.761	3.683	1.334
	East Nusa Tenggara	21.449	16.929	9.209	5.299	3.392	0.904	2.285	1.555	5.596	4.957	7.242	4.145
	Gorontalo	20.861	12.027	11.413	6.480	3.490	2.092	2.790	3.281	4.455	6.265	7.039	4.284
	Jakarta	9.704	5.749	3.640	1.595	1.849	0.385	1.435	0.780	6.079	6.214	2.938	1.242
	Jambi	9.054	12.933	5.674	1.295	5.535	0.917	4.279	1.340	11.166	8.358	5.906	2.418
	Lampung	12.684	6.739	4.810	1.855	2.142	0.301	1.544	0.862	6.300	6.356	3.813	1.495
	Maluku	31.000	20.865	8.394	5.293	2.983	1.655	2.421	2.166	5.020	5.438	8.040	5.150
	North Kalimantan	36.436	13.081	9.929	1.730	10.225	0.267	17.462	0.468	35.891	2.478	14.257	2.247
	North Maluku	22.206	22.064	8.018	5.795	2.745	1.998	1.951	2.945	4.731	6.613	6.394	5.528
	North Sulawesi	0.706	13.174	0.622	2.362	0.678	0.633	1.055	1.334	1.650	7.193	0.747	2.454
	North Sumatra	19.694	12.981	7.356	3.267	3.693	0.783	2.652	1.595	7.380	7.136	6.506	3.025
	Papua	28.927	50.373	7.966	4.188	2.915	0.826	2.110	1.500	6.545	7.491	7.599	8.506
	Riau	18.179	8.209	4.553	2.028	1.445	0.357	1.280	0.855	5.460	6.199	4.214	1.801
	Riau Islands	11.333	7.237	4.723	2.631	1.590	0.473	1.056	1.072	5.244	7.154	3.316	1.802
	South Kalimantan	19.374	2.510	10.676	0.900	9.382	0.322	3.552	0.249	15.740	0.990	10.163	0.659
	South Sulawesi	16.060	14.855	6.219	5.600	1.705	2.807	1.548	6.206	2.680	16.748	4.351	5.507
	South Sumatra	19.082	22.034	6.736	4.262	3.695	0.862	3.054	0.736	9.094	2.568	6.033	3.822
	Southeast Sulawesi	4.942	12.785	3.456	4.022	2.893	1.273	1.828	2.040	3.724	5.353	3.209	3.477
	West Java	10.312	5.297	2.806	1.557	1.728	0.334	1.496	0.569	4.530	2.352	2.867	1.159
	West Kalimantan	12.886	8.362	4.195	2.171	2.388	0.554	1.867	1.141	6.380	6.440	3.980	1.954
	West Nusa Tenggara	16.756	22.142	3.794	5.688	1.608	0.867	1.586	1.578	2.814	3.458	3.864	4.183
	West Papua	18.336	12.390	5.323	3.109	2.291	1.629	1.840	2.620	4.237	6.015	4.986	3.501
	West Sulawesi	29.056	19.891	8.588	4.428	2.620	0.866	1.900	1.697	5.948	7.983	7.586	4.319
	West Sumatra	8.239	9.412	2.260	1.319	1.266	0.528	1.248	0.772	2.222	1.738	2.319	1.725
	Yogyakarta	5.297	2.636	1.905	0.863	1.447	0.429	1.404	1.244	5.081	7.111	2.006	1.232
	**Indonesia**	**13.067**	**9.393**	**4.613**	**2.536**	**2.440**	**0.641**	**1.971**	**1.357**	**5.417**	**4.852**	**3.988**	**2.089**

**2008**	Aceh	14.611	11.566	5.212	2.571	2.661	0.509	1.866	1.085	7.181	6.477	4.643	2.392
	Bali	5.685	2.030	2.712	0.718	2.271	0.345	2.593	0.902	4.775	2.759	2.788	0.734
	Bangka-Belitung Islands	8.498	8.613	4.248	2.533	3.750	1.033	2.190	1.907	7.274	5.830	4.225	2.326
	Banten	16.886	8.448	5.735	2.235	2.425	0.505	1.642	0.982	6.688	7.097	4.623	1.862
	Bengkulu	11.240	5.504	4.365	2.146	2.626	0.946	1.713	1.345	5.441	6.853	3.901	1.862
	Central Java	7.668	6.017	3.111	2.551	1.919	0.637	2.132	1.740	4.232	5.345	2.772	1.814
	Central Kalimantan	17.434	6.418	12.265	2.973	10.358	1.452	12.908	3.875	24.575	15.069	11.949	2.733
	Central Sulawesi	3.920	11.358	0.740	3.767	1.106	1.089	1.144	1.897	2.220	4.852	1.375	2.996
	East Java	12.753	4.872	4.442	1.563	2.264	0.361	1.763	0.786	6.385	2.982	3.557	1.088
	East Kalimantan	8.762	4.496	3.698	1.224	2.744	0.503	1.781	1.204	5.605	6.811	3.555	1.257
	East Nusa Tenggara	20.673	15.838	8.689	4.873	3.195	0.839	2.256	1.515	5.758	4.998	6.881	3.854
	Gorontalo	21.247	12.026	11.101	6.298	3.463	2.042	2.808	3.227	4.535	6.305	6.946	4.191
	Jakarta	9.585	5.579	3.531	1.528	1.790	0.371	1.407	0.764	6.041	6.234	2.862	1.202
	Jambi	8.948	12.041	5.203	1.228	4.710	0.852	3.498	1.262	10.478	8.414	5.229	2.260
	Lampung	13.011	6.752	4.674	1.803	2.102	0.294	1.542	0.843	6.388	6.452	3.774	1.476
	Maluku	30.380	19.162	8.478	4.807	3.055	1.556	2.452	2.088	5.342	5.591	7.984	4.740
	North Kalimantan	34.976	12.577	9.496	1.661	9.951	0.251	17.351	0.455	35.805	2.580	13.789	2.131
	North Maluku	22.244	21.022	8.154	5.464	2.796	1.898	1.977	2.855	5.005	6.838	6.420	5.231
	North Sulawesi	0.700	12.824	0.606	2.306	0.659	0.608	1.018	1.305	1.633	7.160	0.730	2.374
	North Sumatra	19.599	12.737	7.079	3.103	3.605	0.749	2.607	1.536	7.395	7.157	6.346	2.920
	Papua	29.034	50.957	7.802	4.172	3.097	0.863	2.120	1.480	6.566	7.479	7.647	8.555
	Riau	17.755	7.905	4.291	1.875	1.411	0.344	1.281	0.833	5.509	6.219	4.064	1.716
	Riau Islands	10.662	6.670	4.415	2.398	1.509	0.441	1.062	1.053	5.318	7.169	3.151	1.679
	South Kalimantan	16.701	2.771	9.269	1.050	7.742	0.311	3.024	0.297	13.758	1.261	8.545	0.715
	South Sulawesi	16.193	14.271	6.273	5.096	1.942	2.313	1.766	4.905	3.307	14.142	4.507	4.816
	South Sumatra	18.734	20.922	5.941	4.012	3.267	0.880	2.743	0.953	9.208	3.536	5.535	3.681
	Southeast Sulawesi	4.961	12.583	3.346	3.849	2.845	1.200	1.818	1.974	3.725	5.529	3.154	3.357
	West Java	11.714	5.125	3.306	1.459	1.768	0.312	1.502	0.563	4.614	2.445	3.116	1.105
	West Kalimantan	12.137	7.719	3.909	1.998	2.311	0.532	1.846	1.127	6.381	6.476	3.775	1.823

	West Nusa Tenggara	18.619	20.810	4.352	5.163	1.817	0.993	1.718	1.783	3.270	4.285	4.313	4.028
	West Papua	18.273	11.642	5.426	2.945	2.353	1.541	1.831	2.516	4.358	6.087	5.010	3.301
	West Sulawesi	28.178	19.093	8.135	4.197	2.559	0.836	1.890	1.671	5.992	8.060	7.261	4.113
	West Sumatra	8.657	11.322	2.475	1.624	1.330	0.579	1.290	0.917	2.330	2.229	2.441	2.041
	Yogyakarta	5.557	2.749	1.915	0.865	1.442	0.416	1.407	1.210	5.158	7.174	2.026	1.237
	**Indonesia**	**13.351**	**9.142**	**4.621**	**2.387**	**2.404**	**0.605**	**1.965**	**1.254**	**5.559**	**4.779**	**3.983**	**1.993**
**2009**	Aceh	14.333	10.910	5.057	2.468	2.603	0.497	1.848	1.082	7.215	6.585	4.522	2.280
	Bali	5.442	1.905	2.570	0.669	2.255	0.336	2.556	0.879	4.735	2.713	2.722	0.706
	Bangka-Belitung Islands	8.457	8.261	4.133	2.281	3.564	0.940	2.156	1.834	7.256	5.930	4.076	2.174
	Banten	16.415	8.013	5.454	2.082	2.358	0.481	1.634	0.964	6.735	7.175	4.459	1.764
	Bengkulu	11.024	5.198	4.245	2.012	2.535	0.907	1.691	1.317	5.470	6.960	3.784	1.774
	Central Java	8.003	5.588	3.359	2.359	1.994	0.631	2.146	1.660	4.475	5.541	2.888	1.737
	Central Kalimantan	16.955	5.980	12.027	2.818	10.167	1.393	12.950	3.764	24.869	15.097	11.742	2.612
	Central Sulawesi	5.013	10.929	0.818	3.592	1.203	1.037	1.206	1.836	2.570	4.946	1.573	2.857
	East Java	12.648	4.717	4.337	1.499	2.255	0.328	1.753	0.647	6.464	2.634	3.507	1.005
	East Kalimantan	8.385	4.160	3.597	1.155	2.681	0.475	1.752	1.172	5.572	6.834	3.446	1.185
	East Nusa Tenggara	19.520	14.476	8.226	4.502	3.074	0.795	2.226	1.475	5.840	5.016	6.514	3.550
	Gorontalo	21.344	11.835	10.747	6.119	3.364	1.995	2.783	3.202	4.587	6.431	6.763	4.083
	Jakarta	9.313	5.301	3.421	1.464	1.737	0.356	1.379	0.748	5.989	6.235	2.774	1.153
	Jambi	8.728	10.525	4.822	1.171	4.026	0.793	2.923	1.201	9.794	8.484	4.658	2.046
	Lampung	13.179	6.617	4.550	1.720	2.037	0.281	1.532	0.824	6.460	6.549	3.704	1.431
	Maluku	28.663	17.512	8.303	4.534	3.054	1.492	2.471	2.060	5.571	5.799	7.673	4.409
	North Kalimantan	33.746	11.844	9.250	1.598	9.765	0.238	17.270	0.444	35.706	2.638	13.441	1.988
	North Maluku	21.631	19.609	8.016	5.078	2.712	1.775	1.962	2.759	5.149	6.960	6.218	4.860
	North Sulawesi	0.702	12.101	0.595	2.234	0.631	0.578	0.977	1.270	1.610	7.133	0.708	2.254
	North Sumatra	19.641	12.463	6.901	2.982	3.561	0.720	2.579	1.481	7.430	7.193	6.250	2.824
	Papua	28.726	49.527	7.620	4.054	3.083	0.838	2.111	1.443	6.562	7.415	7.518	8.260
	Riau	17.294	7.505	4.122	1.770	1.389	0.334	1.276	0.814	5.556	6.259	3.933	1.631
	Riau Islands	10.085	6.184	4.195	2.228	1.446	0.416	1.058	1.044	5.369	7.170	3.019	1.580

	South Kalimantan	14.336	2.823	8.223	1.142	6.564	0.305	2.735	0.349	12.339	1.570	7.337	0.743
	South Sulawesi	15.840	13.481	6.193	4.713	2.118	1.968	1.936	4.058	3.893	12.488	4.539	4.294
	South Sumatra	18.518	20.168	5.504	3.898	3.024	0.888	2.549	1.114	9.351	4.323	5.241	3.592
	Southeast Sulawesi	4.943	12.129	3.255	3.659	2.794	1.125	1.795	1.903	3.713	5.627	3.095	3.197
	West Java	12.130	4.769	3.594	1.363	1.780	0.296	1.506	0.553	4.678	2.506	3.202	1.035
	West Kalimantan	11.371	6.973	3.692	1.859	2.234	0.509	1.809	1.114	6.368	6.512	3.580	1.690
	West Nusa Tenggara	19.333	19.333	4.716	4.751	1.960	1.082	1.809	1.950	3.656	5.068	4.543	3.859
	West Papua	17.625	10.660	5.302	2.677	2.184	1.397	1.788	2.414	4.375	6.077	4.778	3.016
	West Sulawesi	26.746	17.577	7.715	3.929	2.472	0.798	1.856	1.642	6.014	8.156	6.852	3.805
	West Sumatra	8.474	11.743	2.550	1.812	1.353	0.603	1.302	1.012	2.404	2.656	2.435	2.147
	Yogyakarta	5.622	2.733	1.909	0.860	1.436	0.403	1.411	1.170	5.236	7.217	2.027	1.230
	**Indonesia**	**13.293**	**8.718**	**4.590**	**2.258**	**2.365**	**0.575**	**1.953**	**1.176**	**5.662**	**4.743**	**3.930**	**1.890**
**2010**	Aceh	13.615	9.944	4.862	2.329	2.532	0.483	1.830	1.077	7.268	6.709	4.335	2.126
	Bali	5.406	1.707	2.474	0.626	2.253	0.334	2.538	0.854	4.781	2.685	2.695	0.677
	Bangka-Belitung Islands	7.944	6.901	3.686	1.380	2.709	0.705	2.051	1.705	7.144	5.946	3.420	1.704
	Banten	15.659	7.385	5.182	1.942	2.291	0.460	1.635	0.946	6.767	7.230	4.267	1.649
	Bengkulu	10.296	4.608	4.033	1.826	2.399	0.858	1.662	1.281	5.491	7.058	3.567	1.638
	Central Java	8.033	5.080	3.484	2.180	2.038	0.623	2.157	1.600	4.677	5.722	2.935	1.658
	Central Kalimantan	16.115	5.350	11.581	2.614	9.678	1.302	12.855	3.647	25.090	15.098	11.269	2.442
	Central Sulawesi	5.990	9.971	0.859	3.186	1.201	0.915	1.247	1.745	2.932	5.017	1.688	2.566
	East Java	12.275	4.411	4.202	1.416	2.239	0.302	1.738	0.544	6.517	2.360	3.426	0.920
	East Kalimantan	7.933	3.787	3.458	1.096	2.628	0.459	1.721	1.142	5.524	6.829	3.326	1.117
	East Nusa Tenggara	17.930	12.393	7.560	3.893	2.811	0.714	2.180	1.438	5.921	5.090	5.969	3.087
	Gorontalo	21.151	10.657	10.447	5.650	3.459	1.831	2.871	2.923	4.686	5.893	6.679	3.709
	Jakarta	8.912	4.888	3.299	1.385	1.677	0.342	1.346	0.734	5.925	6.238	2.667	1.089
	Jambi	8.490	9.168	4.513	1.106	3.515	0.743	2.527	1.148	9.235	8.580	4.215	1.855
	Lampung	13.080	6.267	4.393	1.628	1.997	0.275	1.525	0.808	6.526	6.665	3.614	1.368
	Maluku	26.359	15.059	7.871	3.983	2.848	1.343	2.423	1.988	5.737	5.931	7.112	3.862
	North Kalimantan	32.702	11.101	9.047	1.537	9.546	0.226	17.073	0.433	35.547	2.697	13.095	1.847

	North Maluku	20.758	17.834	7.716	4.719	2.618	1.692	1.928	2.683	5.249	7.105	5.938	4.478
	North Sulawesi	0.699	11.777	0.581	2.194	0.616	0.560	0.946	1.240	1.596	7.142	0.694	2.183
	North Sumatra	19.413	11.844	6.719	2.846	3.508	0.693	2.556	1.432	7.465	7.229	6.111	2.684
	Papua	27.349	44.939	7.326	3.748	2.791	0.730	2.043	1.361	6.476	7.267	7.057	7.437
	Riau	16.477	6.876	3.934	1.652	1.362	0.324	1.270	0.795	5.618	6.322	3.750	1.515
	Riau Islands	9.460	5.500	3.998	2.063	1.362	0.385	1.056	1.031	5.404	7.147	2.866	1.449
	South Kalimantan	11.945	2.693	7.170	1.168	5.541	0.297	2.532	0.406	11.170	1.937	6.237	0.742
	South Sulawesi	15.225	12.319	6.025	4.333	2.248	1.665	2.086	3.402	4.489	11.147	4.499	3.793
	South Sumatra	17.979	18.825	5.133	3.712	2.828	0.895	2.398	1.252	9.465	5.053	4.955	3.423
	Southeast Sulawesi	4.845	11.370	3.146	3.443	2.730	1.061	1.786	1.842	3.729	5.755	3.017	3.000
	West Java	11.959	4.268	3.738	1.248	1.766	0.279	1.513	0.541	4.735	2.542	3.183	0.947
	West Kalimantan	10.427	6.020	3.459	1.711	2.153	0.485	1.773	1.098	6.340	6.545	3.362	1.533
	West Nusa Tenggara	18.947	17.244	4.882	4.305	2.056	1.155	1.871	2.111	3.999	5.868	4.572	3.619
	West Papua	14.943	8.274	4.308	1.805	1.347	0.995	1.587	2.193	4.272	5.978	3.714	2.258
	West Sulawesi	25.163	15.854	7.295	3.675	2.394	0.768	1.827	1.609	6.025	8.273	6.432	3.484
	West Sumatra	8.012	11.314	2.542	1.921	1.370	0.616	1.318	1.089	2.468	3.071	2.377	2.138
	Yogyakarta	5.634	2.650	1.897	0.846	1.431	0.390	1.412	1.132	5.307	7.274	2.021	1.217
	**Indonesia**	**12.882**	**7.997**	**4.486**	**2.096**	**2.303**	**0.543**	**1.941**	**1.114**	**5.751**	**4.729**	**3.813**	**1.754**
**2011**	Aceh	12.957	8.622	4.610	2.052	2.293	0.439	1.776	1.058	7.233	6.721	4.044	1.884
	Bali	5.174	1.599	2.191	0.570	2.134	0.321	2.394	0.835	4.677	2.780	2.530	0.655
	Bangka-Belitung Islands	8.113	7.194	3.891	2.106	3.420	0.863	2.092	1.725	7.189	6.059	3.879	1.962
	Banten	15.080	6.765	4.934	1.799	2.220	0.439	1.616	0.922	6.772	7.210	4.093	1.534
	Bengkulu	9.659	4.005	3.796	1.583	2.164	0.782	1.598	1.228	5.442	7.058	3.294	1.472
	Central Java	7.983	4.510	3.481	1.943	1.999	0.572	2.066	1.448	4.812	5.618	2.888	1.514
	Central Kalimantan	15.484	4.727	11.180	2.446	9.552	1.266	12.821	3.576	25.372	15.078	11.051	2.318
	Central Sulawesi	6.960	9.570	1.031	3.227	1.368	0.944	1.301	1.717	3.299	5.106	1.918	2.525
	East Java	12.016	4.049	4.082	1.326	2.207	0.276	1.713	0.452	6.570	2.029	3.346	0.829
	East Kalimantan	7.464	3.346	3.310	1.005	2.523	0.430	1.677	1.104	5.471	6.767	3.170	1.026
	East Nusa Tenggara	16.939	11.394	7.206	3.703	2.853	0.718	2.210	1.430	5.991	5.097	5.735	2.901

	Gorontalo	21.250	9.631	9.817	5.080	3.376	1.642	2.905	2.670	4.822	5.472	6.449	3.324
	Jakarta	8.555	4.504	3.169	1.312	1.613	0.327	1.312	0.719	5.894	6.201	2.560	1.028
	Jambi	8.244	9.125	4.278	1.051	3.245	0.721	2.287	1.111	8.776	8.625	3.943	1.814
	Lampung	13.091	5.890	4.271	1.536	1.948	0.266	1.510	0.790	6.612	6.739	3.536	1.301
	Maluku	25.071	13.423	7.519	3.778	2.859	1.327	2.381	1.953	5.858	6.033	6.809	3.573
	North Kalimantan	31.867	10.468	8.860	1.499	9.328	0.219	16.897	0.422	35.365	2.718	12.785	1.729
	North Maluku	20.764	16.772	7.577	4.595	2.695	1.679	1.894	2.629	5.290	7.210	5.887	4.275
	North Sulawesi	0.693	11.105	0.566	2.121	0.586	0.538	0.904	1.211	1.573	7.103	0.668	2.073
	North Sumatra	19.241	11.098	6.578	2.698	3.437	0.660	2.523	1.374	7.406	7.104	5.975	2.520
	Papua	26.476	41.727	7.031	3.525	2.639	0.673	1.995	1.304	6.397	7.091	6.737	6.847
	Riau	15.793	6.269	3.749	1.535	1.324	0.311	1.249	0.773	5.650	6.328	3.577	1.400
	Riau Islands	8.897	4.860	3.803	1.911	1.280	0.355	1.035	1.012	5.365	7.001	2.715	1.323
	South Kalimantan	10.017	2.490	6.288	1.165	4.731	0.290	2.359	0.463	10.183	2.337	5.354	0.731
	South Sulawesi	14.912	11.384	5.950	4.068	2.394	1.443	2.252	2.888	5.083	9.923	4.525	3.403
	South Sumatra	17.895	17.872	4.896	3.618	2.654	0.905	2.277	1.397	9.513	5.796	4.756	3.318
	Southeast Sulawesi	4.773	10.759	3.048	3.276	2.686	1.010	1.765	1.781	3.725	5.820	2.953	2.838
	West Java	11.868	3.815	3.901	1.152	1.757	0.264	1.514	0.520	4.748	2.467	3.177	0.865
	West Kalimantan	9.533	4.868	3.218	1.495	1.998	0.444	1.709	1.073	6.291	6.499	3.106	1.332
	West Nusa Tenggara	18.074	15.506	4.922	3.957	2.124	1.251	1.904	2.312	4.266	6.755	4.499	3.458
	West Papua	15.686	8.446	4.844	2.219	1.850	1.189	1.624	2.237	4.284	6.014	4.187	2.480
	West Sulawesi	24.007	14.390	6.847	3.431	2.313	0.740	1.788	1.581	6.008	8.341	6.059	3.202
	West Sumatra	7.312	10.527	2.474	1.989	1.355	0.623	1.309	1.160	2.486	3.469	2.261	2.081
	Yogyakarta	5.611	2.533	1.877	0.829	1.414	0.377	1.403	1.093	5.342	7.264	2.004	1.198
	**Indonesia**	**12.588**	**7.373**	**4.398**	**1.965**	**2.252**	**0.516**	**1.908**	**1.045**	**5.808**	**4.616**	**3.714**	**1.633**
**2012**	Aceh	12.925	8.533	4.601	2.145	2.399	0.461	1.761	1.064	7.305	6.845	4.069	1.895
	Bali	4.951	1.450	1.949	0.508	2.016	0.312	2.210	0.814	4.605	2.916	2.369	0.633
	Bangka-Belitung Islands	8.393	7.026	3.906	2.279	3.564	0.845	2.084	1.658	7.258	6.442	3.978	1.961
	Banten	14.877	6.326	4.789	1.699	2.168	0.421	1.609	0.907	6.802	7.230	3.989	1.448
	Bengkulu	9.740	3.941	3.721	1.674	2.278	0.803	1.603	1.237	5.417	7.180	3.334	1.495

	Central Java	7.900	4.109	3.299	1.735	1.904	0.514	1.929	1.325	4.929	5.750	2.765	1.402
	Central Kalimantan	14.824	4.206	10.721	2.284	9.434	1.223	12.759	3.496	25.692	15.092	10.820	2.204
	Central Sulawesi	7.900	8.605	1.134	2.791	1.353	0.799	1.312	1.616	3.638	5.387	2.021	2.222
	East Java	12.140	4.068	4.021	1.269	2.139	0.271	1.672	0.487	6.591	2.561	3.279	0.847
	East Kalimantan	7.170	3.088	3.197	0.959	2.484	0.420	1.648	1.076	5.462	6.773	3.079	0.976
	East Nusa Tenggara	17.455	11.809	6.945	3.717	2.846	0.734	2.073	1.377	6.127	5.434	5.669	2.936
	Gorontalo	21.704	9.463	9.625	4.940	3.294	1.585	2.880	2.667	4.856	5.610	6.340	3.226
	Jakarta	8.439	4.262	3.083	1.257	1.561	0.315	1.281	0.708	5.862	6.224	2.490	0.987
	Jambi	8.258	8.009	4.094	0.997	2.901	0.670	2.055	1.064	8.344	8.698	3.663	1.650
	Lampung	13.224	5.578	4.169	1.461	1.901	0.256	1.501	0.771	6.745	6.850	3.473	1.243
	Maluku	23.945	11.710	7.075	3.360	2.766	1.197	2.283	1.886	6.140	6.487	6.447	3.175
	North Kalimantan	31.536	9.659	8.746	1.388	9.128	0.198	16.687	0.400	35.277	2.886	12.553	1.571
	North Maluku	20.438	15.045	7.149	4.070	2.506	1.490	1.795	2.522	5.494	7.639	5.580	3.811
	North Sulawesi	0.708	11.074	0.552	2.098	0.569	0.521	0.875	1.183	1.557	7.113	0.655	2.031
	North Sumatra	18.754	10.989	6.163	2.560	3.322	0.619	2.398	1.336	7.734	7.775	5.711	2.449
	Papua	26.531	39.441	6.922	3.338	2.502	0.613	1.965	1.255	6.348	6.974	6.583	6.382
	Riau	15.266	5.764	3.614	1.437	1.296	0.299	1.235	0.754	5.694	6.381	3.437	1.303
	Riau Islands	8.541	4.667	3.665	1.837	1.288	0.357	1.031	1.003	5.366	6.919	2.651	1.284
	South Kalimantan	8.655	2.370	5.608	1.177	4.124	0.290	2.251	0.525	9.422	2.787	4.699	0.737
	South Sulawesi	13.810	9.739	5.396	3.500	2.277	1.264	2.129	2.548	5.383	9.682	4.185	2.971
	South Sumatra	17.034	15.802	4.477	3.099	2.422	0.766	2.082	1.245	9.859	5.710	4.405	2.881
	Southeast Sulawesi	4.802	10.026	2.983	2.984	2.644	0.901	1.757	1.689	3.744	6.131	2.911	2.606
	West Java	11.445	3.831	3.731	1.127	1.660	0.257	1.434	0.539	4.906	2.925	3.017	0.867
	West Kalimantan	8.961	4.348	3.044	1.407	1.939	0.430	1.671	1.062	6.285	6.542	2.956	1.246
	West Nusa Tenggara	17.237	12.384	4.732	3.198	2.068	1.039	1.850	2.061	4.552	6.670	4.305	2.836
	West Papua	15.353	7.624	4.599	1.968	1.741	1.067	1.548	2.162	4.401	6.264	4.001	2.239
	West Sulawesi	23.565	13.494	6.585	3.253	2.243	0.706	1.749	1.553	6.008	8.470	5.822	3.003
	West Sumatra	7.670	8.806	2.471	1.790	1.341	0.558	1.298	1.079	2.669	3.588	2.276	1.811
	Yogyakarta	5.722	2.470	1.882	0.817	1.401	0.362	1.410	1.057	5.448	7.335	2.006	1.186

	**Indonesia**	**12.365**	**6.930**	**4.213**	**1.826**	**2.165**	**0.479**	**1.833**	**1.002**	**5.910**	**4.897**	**3.577**	**1.540**
**2013**	Aceh	12.368	8.014	4.485	2.083	2.313	0.455	1.721	1.048	7.156	6.891	3.899	1.808
	Bali	4.575	1.274	1.710	0.446	1.905	0.303	2.038	0.790	4.523	3.083	2.202	0.611
	Bangka-Belitung Islands	8.889	6.842	4.017	2.221	3.428	0.785	2.054	1.571	7.165	6.663	3.944	1.882
	Banten	14.776	5.995	4.632	1.604	2.104	0.405	1.589	0.886	6.770	7.174	3.882	1.374
	Bengkulu	9.780	3.766	3.727	1.714	2.366	0.806	1.586	1.217	5.688	7.245	3.372	1.482
	Central Java	7.983	3.833	3.160	1.545	1.829	0.461	1.818	1.203	5.052	5.834	2.678	1.303
	Central Kalimantan	15.056	3.987	10.474	2.186	9.417	1.202	12.685	3.400	25.876	14.981	10.777	2.144
	Central Sulawesi	8.796	8.102	1.429	2.772	1.554	0.802	1.357	1.548	3.964	5.557	2.276	2.144
	East Java	12.173	3.984	3.918	1.213	2.052	0.265	1.620	0.505	6.573	3.032	3.182	0.853
	East Kalimantan	6.896	2.847	3.070	0.911	2.411	0.406	1.616	1.046	5.448	6.741	2.966	0.927
	East Nusa Tenggara	16.698	10.665	6.308	3.215	2.559	0.649	1.913	1.297	6.176	5.680	5.210	2.615
	Gorontalo	22.132	9.228	9.766	4.929	3.425	1.573	2.888	2.627	4.840	5.606	6.418	3.168
	Jakarta	8.193	3.974	2.959	1.179	1.509	0.301	1.252	0.686	5.805	6.159	2.400	0.933
	Jambi	8.183	9.168	3.993	0.965	2.817	0.680	1.958	1.042	8.024	8.763	3.554	1.745
	Lampung	13.167	5.464	4.086	1.391	1.817	0.250	1.467	0.755	6.554	6.917	3.363	1.207
	Maluku	22.574	10.076	6.718	3.059	2.707	1.105	2.201	1.804	6.264	6.752	6.091	2.833
	North Kalimantan	31.927	9.088	8.665	1.307	8.955	0.182	16.469	0.380	34.975	2.967	12.414	1.451
	North Maluku	20.122	13.507	6.887	3.737	2.424	1.354	1.731	2.389	5.591	7.857	5.370	3.442
	North Sulawesi	0.711	11.526	0.539	2.109	0.563	0.518	0.851	1.155	1.531	7.081	0.646	2.036
	North Sumatra	18.791	9.929	5.912	2.435	3.164	0.571	2.268	1.218	7.657	7.614	5.512	2.240
	Papua	27.953	40.567	7.005	3.473	2.736	0.665	1.946	1.251	6.284	6.891	6.844	6.491
	Riau	15.033	5.425	3.486	1.353	1.272	0.291	1.220	0.736	5.717	6.396	3.332	1.230
	Riau Islands	8.362	4.407	3.568	1.753	1.259	0.339	1.014	0.980	5.364	6.796	2.586	1.219
	South Kalimantan	7.560	2.240	5.029	1.176	3.649	0.294	2.146	0.585	8.724	3.253	4.171	0.744
	South Sulawesi	12.951	8.404	4.950	3.033	2.186	1.084	2.043	2.212	5.687	9.223	3.918	2.586
	South Sumatra	16.780	14.002	4.238	2.645	2.228	0.652	1.959	1.129	10.643	5.751	4.188	2.511
	Southeast Sulawesi	4.834	9.345	2.939	2.796	2.586	0.827	1.711	1.591	3.708	6.286	2.860	2.419
	West Java	11.003	3.713	3.590	1.080	1.578	0.249	1.367	0.551	4.995	3.311	2.870	0.850

	West Kalimantan	8.520	3.987	2.891	1.354	1.938	0.427	1.659	1.047	6.294	6.521	2.860	1.189
	West Nusa Tenggara	16.877	10.301	4.691	2.705	2.061	0.899	1.805	1.857	4.818	6.658	4.219	2.422
	West Papua	15.292	7.106	4.682	2.015	2.054	1.102	1.544	2.099	4.495	6.385	4.162	2.191
	West Sulawesi	22.875	12.483	6.402	3.095	2.203	0.680	1.711	1.516	5.970	8.514	5.591	2.801
	West Sumatra	7.835	7.296	2.464	1.618	1.345	0.513	1.298	1.030	2.870	3.796	2.279	1.592
	Yogyakarta	5.739	2.367	1.871	0.800	1.370	0.348	1.391	1.024	5.485	7.395	1.980	1.171
	**Indonesia**	**12.210**	**6.519**	**4.060**	**1.699**	**2.087**	**0.449**	**1.765**	**0.952**	**5.968**	**5.091**	**3.456**	**1.452**
**2014**	Aceh	12.067	7.583	4.318	2.005	2.221	0.419	1.691	1.032	7.077	6.944	3.746	1.726
	Bali	4.234	1.141	1.499	0.397	1.793	0.289	1.871	0.764	4.459	3.284	2.045	0.598
	Bangka-Belitung Islands	9.513	6.885	4.176	2.164	3.292	0.681	2.028	1.490	7.077	6.875	3.928	1.827
	Banten	14.741	5.841	4.584	1.567	2.073	0.368	1.569	0.864	6.756	7.102	3.820	1.331
	Bengkulu	9.231	3.396	3.601	1.614	2.334	0.740	1.565	1.185	5.895	7.281	3.255	1.398
	Central Java	7.772	3.627	3.063	1.395	1.776	0.378	1.707	1.083	5.212	5.938	2.587	1.221
	Central Kalimantan	16.726	4.192	10.839	2.230	9.283	1.157	12.501	3.288	25.906	14.912	10.906	2.155
	Central Sulawesi	9.381	7.419	1.704	2.527	1.638	0.687	1.377	1.466	4.313	5.735	2.426	1.964
	East Java	12.107	4.018	3.854	1.166	2.003	0.242	1.577	0.520	6.540	3.574	3.105	0.872
	East Kalimantan	6.287	2.511	2.915	0.836	2.363	0.373	1.580	1.010	5.393	6.667	2.828	0.859
	East Nusa Tenggara	15.519	9.574	5.680	2.737	2.326	0.582	1.784	1.219	6.255	6.011	4.736	2.317
	Gorontalo	22.205	8.960	9.622	4.776	3.392	1.455	2.853	2.568	4.840	5.602	6.307	3.051
	Jakarta	7.716	3.652	2.830	1.106	1.472	0.265	1.231	0.666	5.773	6.052	2.298	0.877
	Jambi	8.808	8.851	3.852	0.934	2.617	0.628	1.819	1.007	7.658	8.779	3.427	1.676
	Lampung	13.579	5.451	4.043	1.345	1.751	0.236	1.408	0.735	6.362	6.934	3.306	1.182
	Maluku	21.053	8.775	6.288	2.701	2.585	0.939	2.109	1.721	6.317	7.051	5.668	2.515
	North Kalimantan	32.547	8.879	8.731	1.248	8.822	0.158	16.204	0.360	34.517	3.046	12.342	1.379
	North Maluku	19.606	12.282	6.615	3.404	2.355	1.152	1.672	2.279	5.634	8.106	5.141	3.132
	North Sulawesi	0.725	11.644	0.532	2.078	0.552	0.496	0.830	1.126	1.519	7.073	0.638	2.001
	North Sumatra	18.459	9.230	5.684	2.250	3.008	0.498	2.149	1.114	7.628	7.552	5.277	2.068
	Papua	28.109	39.024	6.991	3.363	2.633	0.618	1.886	1.200	6.194	6.698	6.719	6.131
	Riau	14.863	5.143	3.406	1.286	1.254	0.272	1.205	0.717	5.719	6.432	3.245	1.168

	Riau Islands	8.232	4.218	3.444	1.602	1.214	0.325	1.002	0.962	5.366	6.698	2.508	1.160
	South Kalimantan	6.690	2.111	4.566	1.168	3.232	0.302	2.060	0.641	8.157	3.719	3.729	0.750
	South Sulawesi	11.690	7.054	4.549	2.589	2.130	0.890	1.974	1.856	6.069	8.668	3.649	2.200
	South Sumatra	15.788	12.075	4.025	2.245	2.098	0.469	1.834	1.028	9.558	5.845	3.901	2.160
	Southeast Sulawesi	5.001	9.148	2.970	2.692	2.543	0.718	1.673	1.503	3.681	6.486	2.849	2.317
	West Java	10.393	3.613	3.384	1.052	1.492	0.227	1.298	0.557	5.061	3.707	2.694	0.837
	West Kalimantan	7.754	3.413	2.701	1.244	1.893	0.394	1.626	1.025	6.258	6.446	2.699	1.092
	West Nusa Tenggara	15.858	8.353	4.578	2.223	2.039	0.666	1.749	1.654	5.089	6.612	4.040	2.030
	West Papua	14.673	6.401	4.534	1.825	1.934	0.976	1.465	1.970	4.483	6.445	3.950	1.977
	West Sulawesi	22.030	11.782	5.976	2.946	2.151	0.620	1.684	1.482	5.988	8.615	5.283	2.640
	West Sumatra	7.725	5.881	2.448	1.444	1.342	0.424	1.294	0.985	3.101	4.040	2.248	1.388
	Yogyakarta	5.548	2.221	1.817	0.767	1.346	0.322	1.371	0.998	5.499	7.416	1.935	1.150
	**Indonesia**	**11.878**	**6.110**	**3.916**	**1.574**	**2.012**	**0.392**	**1.697**	**0.901**	**5.984**	**5.307**	**3.320**	**1.365**
**2015**	Aceh	11.802	6.865	4.146	1.857	2.064	1.032	1.634	1.005	6.884	7.047	3.554	1.594
	Bali	3.592	0.951	1.239	0.341	1.678	0.764	1.674	0.739	4.322	3.535	1.849	0.585
	Bangka-Belitung Islands	9.712	6.934	4.131	2.101	3.186	1.490	1.987	1.435	7.121	7.047	3.849	1.775
	Banten	14.987	5.461	4.366	1.460	1.902	0.864	1.516	0.840	6.564	7.080	3.660	1.246
	Bengkulu	8.530	3.074	3.391	1.542	2.252	1.185	1.543	1.163	5.811	7.278	3.075	1.330
	Central Java	7.774	3.344	2.859	1.269	1.658	1.083	1.631	1.028	5.179	6.040	2.458	1.155
	Central Kalimantan	16.483	4.151	10.781	2.194	9.149	3.288	12.298	3.204	25.733	14.798	10.762	2.123
	Central Sulawesi	9.578	6.961	1.669	2.326	1.586	1.466	1.348	1.417	4.278	5.877	2.380	1.829
	East Java	12.043	3.711	3.605	1.068	1.805	0.520	1.506	0.518	6.414	3.820	2.908	0.837
	East Kalimantan	6.409	2.446	2.800	0.822	2.133	1.010	1.513	0.978	5.246	6.646	2.662	0.836
	East Nusa Tenggara	15.202	8.933	5.215	2.634	2.215	1.219	1.723	1.208	6.254	5.869	4.472	2.198
	Gorontalo	21.418	8.456	9.366	4.589	3.349	2.568	2.829	2.510	4.835	5.581	6.095	2.905
	Jakarta	7.869	3.481	2.663	1.031	1.345	0.666	1.183	0.648	5.606	6.097	2.186	0.835
	Jambi	8.356	8.118	3.760	0.880	2.606	1.007	1.814	0.981	7.708	8.772	3.343	1.568
	Lampung	14.150	5.299	3.918	1.280	1.629	0.735	1.354	0.716	6.210	6.872	3.214	1.141
	Maluku	20.143	7.740	5.884	2.454	2.465	1.721	2.043	1.674	6.244	7.271	5.329	2.289

	North Kalimantan	31.734	8.451	8.637	1.179	8.666	0.360	15.946	0.348	34.168	3.084	12.089	1.284
	North Maluku	19.476	11.159	6.261	3.088	2.232	2.279	1.626	2.208	5.585	8.315	4.905	2.855
	North Sulawesi	0.726	11.477	0.523	2.050	0.534	1.126	0.804	1.113	1.497	7.230	0.623	1.961
	North Sumatra	18.689	8.996	5.396	2.136	2.800	1.114	2.058	1.063	7.514	7.513	5.063	1.975
	Papua	29.347	39.663	6.909	3.409	2.508	1.200	1.826	1.172	5.993	6.599	6.684	6.095
	Riau	13.655	4.496	3.221	1.183	1.223	0.717	1.186	0.700	5.694	6.415	3.017	1.057
	Riau Islands	7.923	4.000	3.223	1.560	1.103	0.962	0.961	0.934	5.176	6.553	2.342	1.107
	South Kalimantan	5.611	2.339	4.081	1.185	2.889	0.641	1.990	0.701	7.642	4.308	3.315	0.798
	South Sulawesi	10.918	6.395	4.135	2.454	1.966	1.856	1.859	1.887	5.980	8.744	3.354	2.113
	South Sumatra	15.338	10.536	3.808	1.909	1.966	1.028	1.761	0.937	9.091	5.944	3.685	1.872
	Southeast Sulawesi	4.905	8.737	2.905	2.530	2.461	1.503	1.630	1.443	3.648	6.620	2.764	2.182
	West Java	10.201	3.386	3.124	0.974	1.369	0.557	1.244	0.550	4.973	3.896	2.523	0.794
	West Kalimantan	7.288	3.109	2.568	1.172	1.841	1.025	1.589	1.008	6.226	6.370	2.577	1.033
	West Nusa Tenggara	16.062	7.275	4.412	1.948	1.921	1.654	1.688	1.516	5.156	6.754	3.918	1.801
	West Papua	14.646	5.973	4.408	1.756	1.989	1.970	1.436	1.927	4.441	6.575	3.916	1.893
	West Sulawesi	22.423	10.607	5.747	2.717	1.996	1.482	1.625	1.460	5.854	8.644	5.100	2.414
	West Sumatra	7.769	4.944	2.484	1.320	1.382	0.985	1.307	0.951	3.443	4.406	2.271	1.259
	Yogyakarta	5.433	2.189	1.807	0.766	1.314	0.998	1.336	0.962	5.445	7.397	1.890	1.133
	**Indonesia**	**11.776**	**5.756**	**3.698**	**1.469**	**1.879**	**0.901**	**1.632**	**0.875**	**5.894**	**5.445**	**3.157**	**1.294**
**2016**	Aceh	10.783	6.309	4.030	1.784	2.074	0.411	1.637	0.989	6.930	7.061	3.404	1.506
	Bali	1.995	0.525	0.850	0.249	1.698	0.287	1.603	0.691	3.912	3.331	1.653	0.527
	Bangka-Belitung Islands	9.518	6.913	4.112	2.106	3.101	0.668	1.969	1.411	7.070	6.877	3.755	1.744
	Banten	13.510	4.940	4.205	1.381	1.882	0.357	1.501	0.819	6.554	7.022	3.444	1.162
	Bengkulu	7.687	2.721	3.225	1.438	2.193	0.705	1.523	1.134	5.755	7.225	2.908	1.251
	Central Java	6.975	3.029	2.679	1.204	1.641	0.372	1.643	1.033	5.136	5.939	2.347	1.116
	Central Kalimantan	15.350	3.854	10.361	2.073	9.019	1.122	12.148	3.121	25.607	14.625	10.482	2.045
	Central Sulawesi	8.946	6.808	1.572	2.308	1.544	0.678	1.310	1.391	4.164	5.781	2.248	1.783
	East Java	10.431	3.205	3.414	0.993	1.804	0.235	1.497	0.505	6.398	3.683	2.746	0.778
	East Kalimantan	6.997	2.733	3.011	0.867	2.018	0.347	1.513	0.951	5.340	6.641	2.683	0.854

	East Nusa Tenggara	13.556	7.869	4.971	2.417	2.209	0.558	1.728	1.185	6.280	5.879	4.181	1.998
	Gorontalo	19.411	7.669	8.870	4.280	3.287	1.386	2.799	2.459	4.829	5.575	5.726	2.712
	Jakarta	7.314	3.277	2.584	0.990	1.325	0.256	1.162	0.631	5.557	5.989	2.096	0.801
	Jambi	7.638	7.449	3.598	0.835	2.599	0.606	1.817	0.965	7.764	8.744	3.230	1.474
	Lampung	13.306	5.056	3.872	1.245	1.634	0.230	1.357	0.699	6.207	6.859	3.102	1.100
	Maluku	17.837	6.871	5.525	2.286	2.428	0.901	2.029	1.658	6.181	7.178	4.902	2.110
	North Kalimantan	29.055	7.741	8.386	1.134	8.469	0.155	15.614	0.339	33.734	3.022	11.612	1.174
	North Maluku	17.810	10.226	5.959	2.907	2.208	1.119	1.615	2.173	5.521	8.187	4.589	2.665
	North Sulawesi	0.668	10.494	0.507	1.928	0.529	0.479	0.804	1.094	1.501	7.251	0.615	1.839
	North Sumatra	18.331	8.909	5.376	2.124	2.798	0.494	2.050	1.057	7.470	7.495	4.967	1.940
	Papua	27.556	37.840	6.731	3.316	2.580	0.626	1.801	1.149	5.895	6.505	6.379	5.731
	Riau	10.532	3.314	2.834	0.997	1.185	0.251	1.176	0.683	5.707	6.324	2.552	0.870
	Riau Islands	7.473	3.774	3.143	1.504	1.100	0.314	0.958	0.912	5.184	6.451	2.255	1.052
	South Kalimantan	5.296	2.034	4.030	1.096	2.932	0.286	1.968	0.655	7.750	4.085	3.293	0.733
	South Sulawesi	9.555	5.444	3.894	2.253	1.945	0.864	1.844	1.881	5.897	8.755	3.137	1.978
	South Sumatra	14.808	10.532	3.755	1.911	1.963	0.471	1.775	0.937	9.165	5.852	3.594	1.847
	Southeast Sulawesi	4.584	8.188	2.800	2.433	2.415	0.699	1.598	1.413	3.613	6.529	2.667	2.067
	West Java	9.033	2.980	3.002	0.908	1.368	0.219	1.249	0.534	4.936	3.753	2.378	0.732
	West Kalimantan	6.725	2.807	2.459	1.110	1.828	0.386	1.575	0.990	6.216	6.322	2.480	0.982
	West Nusa Tenggara	15.087	7.166	4.329	1.989	1.910	0.675	1.676	1.522	5.076	6.686	3.755	1.793
	West Papua	12.964	5.308	4.153	1.600	1.837	0.894	1.399	1.868	4.368	6.459	3.548	1.718
	West Sulawesi	20.737	9.940	5.541	2.595	1.976	0.594	1.603	1.426	5.843	8.690	4.785	2.274
	West Sumatra	6.469	4.181	2.262	1.212	1.346	0.414	1.277	0.938	3.294	4.217	2.056	1.146
	Yogyakarta	5.481	2.257	1.876	0.797	1.294	0.314	1.337	0.950	5.521	7.520	1.891	1.146
	**Indonesia**	**10.698**	**5.322**	**3.553**	**1.396**	**1.868**	**0.383**	**1.627**	**0.862**	**5.861**	**5.350**	**3.004**	**1.225**
**2017**	Aceh	10.255	5.882	3.946	1.722	2.043	0.400	1.614	0.968	6.902	7.022	3.286	1.430
	Bali	1.146	0.295	0.615	0.192	1.668	0.279	1.541	0.663	3.620	3.258	1.521	0.496
	Bangka-Belitung Islands	9.856	7.068	4.188	2.117	3.022	0.650	1.940	1.386	7.025	6.781	3.722	1.728
	Banten	12.820	4.684	4.130	1.334	1.876	0.348	1.493	0.803	6.518	6.957	3.329	1.112
	Bengkulu	7.324	2.553	3.139	1.388	2.142	0.680	1.512	1.113	5.723	7.172	2.810	1.206

Central Java	6.592	2.855	2.559	1.152	1.625	0.365	1.639	1.027	5.087	5.870	2.276	1.089
Central Kalimantan	15.005	3.752	10.236	2.015	8.944	1.099	11.971	3.062	25.514	14.507	10.359	2.008
Central Sulawesi	8.733	6.708	1.532	2.303	1.517	0.665	1.286	1.367	4.100	5.717	2.181	1.744
East Java	9.847	2.992	3.299	0.950	1.790	0.230	1.480	0.497	6.351	3.617	2.657	0.748
East Kalimantan	7.180	2.843	3.048	0.911	2.014	0.351	1.505	0.943	5.344	6.603	2.688	0.872
East Nusa Tenggara	12.992	7.520	4.833	2.320	2.213	0.552	1.736	1.175	6.310	5.902	4.046	1.916
Gorontalo	18.573	7.262	8.619	4.131	3.237	1.332	2.757	2.408	4.825	5.534	5.518	2.592
Jakarta	7.460	3.332	2.591	0.988	1.315	0.252	1.147	0.618	5.489	5.950	2.087	0.798
Jambi	7.369	6.941	3.506	0.806	2.563	0.583	1.801	0.945	7.767	8.686	3.151	1.397
Lampung	13.472	5.034	3.897	1.234	1.633	0.226	1.351	0.689	6.173	6.830	3.081	1.084
Maluku	16.535	6.335	5.310	2.206	2.427	0.884	2.008	1.632	6.115	7.095	4.648	2.003
North Kalimantan	28.273	7.434	8.309	1.110	8.374	0.152	15.398	0.333	33.447	2.954	11.428	1.112
North Maluku	17.712	9.989	5.995	2.915	2.171	1.088	1.604	2.137	5.483	8.110	4.501	2.592
North Sulawesi	0.680	10.630	0.512	1.931	0.528	0.472	0.798	1.071	1.495	7.188	0.618	1.821
North Sumatra	18.273	8.791	5.324	2.080	2.764	0.482	2.023	1.042	7.404	7.427	4.876	1.889
Papua	26.055	35.247	6.569	3.183	2.474	0.588	1.753	1.111	5.783	6.351	6.017	5.249
Riau	9.659	2.981	2.654	0.917	1.156	0.241	1.152	0.667	5.638	6.190	2.378	0.803
Riau Islands	7.412	3.741	3.102	1.481	1.097	0.307	0.949	0.893	5.164	6.375	2.214	1.026
South Kalimantan	5.261	1.945	4.047	1.066	2.933	0.277	1.934	0.629	7.762	3.997	3.278	0.707
South Sulawesi	8.471	4.720	3.623	2.067	1.920	0.837	1.821	1.869	5.808	8.666	2.943	1.863
South Sumatra	14.502	10.301	3.689	1.876	1.947	0.464	1.773	0.927	9.150	5.786	3.509	1.789
Southeast Sulawesi	4.578	8.118	2.808	2.443	2.396	0.691	1.571	1.386	3.603	6.490	2.645	2.032
West Java	8.371	2.715	2.889	0.856	1.355	0.213	1.239	0.523	4.889	3.658	2.272	0.688
West Kalimantan	6.202	2.549	2.332	1.034	1.792	0.372	1.542	0.967	6.153	6.206	2.368	0.929
West Nusa Tenggara	14.264	6.789	4.183	1.931	1.873	0.661	1.655	1.501	5.049	6.631	3.583	1.727
West Papua	12.594	5.101	4.130	1.602	1.910	0.901	1.403	1.848	4.369	6.438	3.511	1.688
West Sulawesi	19.698	9.382	5.387	2.523	1.953	0.577	1.582	1.396	5.846	8.685	4.558	2.164
West Sumatra	5.741	3.669	2.101	1.121	1.312	0.402	1.242	0.919	3.206	4.081	1.916	1.062
Yogyakarta	6.042	2.498	1.994	0.857	1.272	0.307	1.337	0.941	5.620	7.689	1.929	1.177
**Indonesia**	**10.230**	**5.063**	**3.456**	**1.347**	**1.849**	**0.374**	**1.612**	**0.849**	**5.816**	**5.286**	**2.909**	**1.178**

**2018**	Aceh	10.181	5.686	3.852	1.689	2.015	0.388	1.601	0.953	6.860	7.003	3.214	1.384
	Bali	1.576	0.415	0.685	0.215	1.665	0.275	1.544	0.651	3.662	3.202	1.569	0.507
	Bangka-Belitung Islands	9.096	6.431	3.938	2.008	2.983	0.632	1.898	1.356	6.845	6.623	3.561	1.621
	Banten	12.156	4.424	3.954	1.272	1.837	0.334	1.475	0.790	6.458	6.868	3.180	1.057
	Bengkulu	7.375	2.566	3.045	1.364	2.118	0.659	1.499	1.096	5.670	7.103	2.764	1.186
	Central Java	6.529	2.832	2.431	1.119	1.595	0.356	1.642	1.026	5.056	5.788	2.223	1.073
	Central Kalimantan	14.880	3.696	9.940	1.966	8.856	1.069	11.805	3.003	25.369	14.340	10.217	1.973
	Central Sulawesi	8.407	6.523	1.444	2.224	1.484	0.646	1.266	1.339	4.040	5.655	2.094	1.681
	East Java	9.616	2.933	3.190	0.925	1.774	0.224	1.470	0.491	6.300	3.552	2.594	0.730
	East Kalimantan	6.326	2.465	2.786	0.826	1.970	0.337	1.466	0.916	5.189	6.415	2.510	0.803
	East Nusa Tenggara	13.440	7.718	4.751	2.313	2.211	0.541	1.744	1.163	6.344	5.912	4.032	1.906
	Gorontalo	18.667	7.322	8.348	4.028	3.197	1.285	2.745	2.368	4.813	5.495	5.399	2.527
	Jakarta	7.241	3.216	2.381	0.924	1.271	0.239	1.133	0.612	5.439	5.816	1.993	0.766
	Jambi	7.701	7.111	3.447	0.801	2.537	0.562	1.800	0.934	7.758	8.631	3.136	1.385
	Lampung	12.792	4.726	3.757	1.179	1.616	0.219	1.346	0.678	6.136	6.809	2.956	1.036
	Maluku	16.498	6.368	5.135	2.198	2.421	0.873	1.999	1.611	6.046	6.986	4.545	1.976
	North Kalimantan	27.511	7.305	8.142	1.096	8.199	0.147	14.992	0.326	32.772	2.898	11.161	1.070
	North Maluku	17.420	9.676	5.777	2.818	2.168	1.063	1.590	2.102	5.413	8.000	4.360	2.495
	North Sulawesi	0.675	10.365	0.495	1.872	0.521	0.459	0.792	1.054	1.495	7.148	0.612	1.765
	North Sumatra	17.348	8.306	5.124	2.002	2.722	0.467	1.993	1.030	7.324	7.360	4.665	1.797
	Papua	25.578	34.288	6.350	3.103	2.460	0.580	1.728	1.094	5.683	6.266	5.816	4.999
	Riau	9.852	3.054	2.612	0.912	1.143	0.235	1.139	0.655	5.546	6.146	2.351	0.798
	Riau Islands	7.319	3.683	2.950	1.427	1.086	0.299	0.949	0.873	5.109	6.245	2.140	0.990
	South Kalimantan	4.978	1.783	3.899	1.005	2.883	0.264	1.908	0.607	7.749	3.915	3.185	0.670
	South Sulawesi	8.745	4.879	3.586	2.067	1.898	0.828	1.797	1.853	5.711	8.643	2.921	1.867
	South Sumatra	14.000	9.903	3.555	1.822	1.921	0.452	1.771	0.916	9.087	5.720	3.390	1.712
	Southeast Sulawesi	4.634	8.119	2.737	2.403	2.348	0.673	1.545	1.356	3.586	6.466	2.595	1.988
	West Java	8.458	2.724	2.830	0.844	1.343	0.206	1.235	0.513	4.847	3.573	2.240	0.675
	West Kalimantan	6.108	2.527	2.243	1.004	1.781	0.365	1.528	0.955	6.098	6.132	2.322	0.913
	West Nusa Tenggara	13.358	6.368	3.956	1.838	1.832	0.641	1.646	1.482	5.030	6.590	3.392	1.649

	West Papua	12.970	5.243	4.061	1.609	1.954	0.897	1.393	1.816	4.328	6.398	3.526	1.692
	West Sulawesi	19.234	9.267	5.132	2.438	1.911	0.554	1.566	1.370	5.837	8.686	4.366	2.095
	West Sumatra	6.043	3.850	2.054	1.121	1.298	0.392	1.230	0.904	3.153	4.007	1.909	1.060
	Yogyakarta	5.742	2.345	1.850	0.798	1.260	0.297	1.311	0.921	5.488	7.472	1.862	1.136
	**Indonesia**	**10.064**	**4.968**	**3.343**	**1.313**	**1.827**	**0.364**	**1.602**	**0.839**	**5.770**	**5.213**	**2.836**	**1.147**
**2019**	Aceh	9.880	5.427	3.732	1.623	1.979	0.377	1.575	0.938	6.812	6.977	3.112	1.328
	Bali	1.592	0.424	0.662	0.210	1.646	0.269	1.526	0.638	3.622	3.159	1.558	0.506
	Bangka-Belitung Islands	8.587	6.032	3.754	1.905	2.918	0.609	1.856	1.329	6.728	6.492	3.424	1.537
	Banten	11.294	4.098	3.772	1.199	1.800	0.320	1.453	0.778	6.377	6.770	3.017	0.997
	Bengkulu	7.125	2.469	2.909	1.304	2.078	0.636	1.474	1.078	5.601	7.018	2.675	1.147
	Central Java	6.309	2.709	2.314	1.069	1.574	0.347	1.632	1.025	4.996	5.688	2.165	1.049
	Central Kalimantan	14.585	3.602	9.635	1.891	8.758	1.038	11.705	2.951	25.186	14.152	10.065	1.931
	Central Sulawesi	8.024	6.273	1.367	2.129	1.448	0.625	1.238	1.313	3.984	5.598	2.006	1.610
	East Java	9.194	2.782	3.058	0.887	1.747	0.218	1.443	0.486	6.236	3.501	2.510	0.708
	East Kalimantan	5.817	2.241	2.628	0.776	1.919	0.325	1.430	0.895	5.115	6.289	2.385	0.759
	East Nusa Tenggara	13.265	7.581	4.579	2.227	2.189	0.525	1.736	1.151	6.345	5.899	3.923	1.845
	Gorontalo	18.189	7.095	7.940	3.824	3.144	1.236	2.713	2.328	4.782	5.449	5.205	2.422
	Jakarta	6.993	3.107	2.198	0.855	1.234	0.228	1.108	0.604	5.361	5.688	1.904	0.736
	Jambi	7.592	6.920	3.333	0.770	2.507	0.542	1.789	0.921	7.746	8.571	3.071	1.340
	Lampung	12.328	4.498	3.647	1.125	1.586	0.211	1.340	0.672	6.119	6.801	2.852	0.998
	Maluku	15.819	6.132	4.891	2.101	2.399	0.847	1.971	1.583	5.974	6.873	4.355	1.896
	North Kalimantan	26.497	7.008	7.966	1.064	8.089	0.143	14.798	0.321	32.433	2.859	10.950	1.014
	North Maluku	16.924	9.296	5.538	2.681	2.146	1.030	1.567	2.063	5.345	7.872	4.191	2.382
	North Sulawesi	0.652	10.024	0.480	1.802	0.508	0.446	0.777	1.036	1.479	7.092	0.600	1.707
	North Sumatra	16.775	7.994	4.970	1.919	2.673	0.454	1.961	1.017	7.233	7.277	4.502	1.725
	Papua	24.459	32.579	6.057	2.940	2.401	0.559	1.685	1.071	5.559	6.135	5.511	4.654
	Riau	9.532	2.966	2.511	0.870	1.122	0.228	1.118	0.644	5.476	6.065	2.258	0.769
	Riau Islands	7.069	3.533	2.799	1.364	1.071	0.290	0.940	0.854	5.078	6.137	2.048	0.943
	South Kalimantan	4.709	1.642	3.770	0.951	2.863	0.256	1.872	0.591	7.694	3.862	3.114	0.640

	South Sulawesi	8.518	4.732	3.465	1.991	1.868	0.813	1.768	1.838	5.627	8.572	2.837	1.830
	South Sumatra	13.504	9.565	3.432	1.755	1.891	0.439	1.763	0.908	9.086	5.665	3.278	1.642
	Southeast Sulawesi	4.572	7.965	2.661	2.327	2.298	0.654	1.510	1.330	3.555	6.439	2.531	1.924
	West Java	8.110	2.591	2.725	0.807	1.326	0.200	1.219	0.504	4.784	3.484	2.162	0.647
	West Kalimantan	5.812	2.393	2.145	0.950	1.752	0.356	1.499	0.940	6.031	6.059	2.245	0.882
	West Nusa Tenggara	12.643	6.050	3.762	1.744	1.797	0.620	1.624	1.461	4.999	6.547	3.232	1.583
	West Papua	12.596	5.071	3.901	1.547	1.933	0.871	1.376	1.777	4.293	6.336	3.406	1.633
	West Sulawesi	18.378	8.881	4.880	2.316	1.869	0.532	1.535	1.342	5.798	8.667	4.144	1.997
	West Sumatra	5.826	3.685	1.954	1.066	1.270	0.381	1.203	0.888	3.094	3.922	1.838	1.019
	Yogyakarta	5.529	2.245	1.769	0.763	1.233	0.287	1.286	0.905	5.415	7.401	1.807	1.113
	**Indonesia**	**9.687**	**4.771**	**3.213**	**1.256**	**1.799**	**0.354**	**1.581**	**0.829**	**5.711**	**5.141**	**2.742**	**1.106**

Supplementary Table S2. The trend of unintentional drowning mortality rates by sex and age group in Indonesia between 2005 and 2019

Age Group		Mortality rates (per 100,000)															Linear Trend Model	R-squared
		2005	2006	2007	2008	2009	2010	2011	2012	2013	2014	2015	2016	2017	2018	2019		
**Under 5**	Male	13.99	13.52	13.07	13.35	13.29	12.88	12.59	12.36	12.21	11.88	11.78	10.70	10.23	10.06	9.69	y = -0.30x + 14.45	R^2 = 0.94
	Female	10.45	9.86	9.39	9.14	8.72	8.00	7.37	6.93	6.52	6.11	5.76	5.32	5.06	4.97	4.77	y = -0.43x + 10.64	R^2 = 0.98
**5-14 years**	Male	5.21	4.94	4.61	4.62	4.59	4.49	4.40	4.21	4.06	3.92	3.70	3.55	3.46	3.34	3.21	y = -0.14x + 5.23	R^2 = 0.98
	Female	2.96	2.74	2.54	2.39	2.26	2.10	1.96	1.83	1.70	1.57	1.47	1.40	1.35	1.31	1.26	y = -0.12x + 2.90	R^2 = 0.97
**15-49 years**	Male	2.63	2.56	2.44	2.40	2.36	2.30	2.25	2.16	2.09	2.01	1.88	1.87	1.85	1.83	1.80	y = -0.06x + 2.66	R^2 = 0.98
	Female	0.71	0.68	0.64	0.61	0.58	0.54	0.52	0.48	0.45	0.42	0.39	0.38	0.37	0.36	0.35	y = -0.03x + 0.72	R^2 = 0.97
**50-69 years**	Male	2.03	2.04	1.97	1.96	1.95	1.94	1.91	1.83	1.76	1.70	1.63	1.63	1.61	1.60	1.58	y = -0.04x + 2.12	R^2 = 0.95
	Female	1.44	1.42	1.36	1.25	1.18	1.11	1.05	1.00	0.95	0.90	0.88	0.86	0.85	0.84	0.83	y = -0.05x + 1.44	R^2 = 0.93
**70+ years**	Male	5.38	5.44	5.42	5.56	5.66	5.75	5.81	5.91	5.97	5.98	5.89	5.86	5.82	5.77	5.71	y = 0.03x + 5.48	R^2 = 0.50
	Female	4.79	4.83	4.85	4.78	4.74	4.73	4.62	4.90	5.09	5.31	5.44	5.35	5.29	5.21	5.14	y = 0.05x + 4.64	R^2 = 0.59
**All Ages**	Male	4.35	4.19	3.99	3.98	3.93	3.81	3.71	3.58	3.46	3.32	3.16	3.00	2.91	2.84	2.74	y = -0.12x + 4.45	R^2 = 0.99
	Female	2.34	2.21	2.09	1.99	1.89	1.75	1.63	1.54	1.45	1.36	1.29	1.23	1.18	1.15	1.11	y = -0.09 + 2.34	R^2 = 0.97
